# Synthesis, Biocidal and Antibiofilm Activities of New Isatin–Quinoline Conjugates against Multidrug-Resistant Bacterial Pathogens along with Their In Silico Screening

**DOI:** 10.3390/antibiotics11111507

**Published:** 2022-10-28

**Authors:** Elshaymaa I. Elmongy, Abdullah A. S. Ahmed, Ibrahim El Tantawy El Sayed, Ghady Fathy, Hanem M. Awad, Ayah Usama Salman, Mohamed A. Hamed

**Affiliations:** 1 Department of Pharmaceutical Sciences, College of Pharmacy, Princess Nourah bint Abdulrahman University, P.O. Box 84428, Riyadh 11671, Saudi Arabia; 2 Chemistry Department, Faculty of Science, Menoufia University, Shebin El-Kom 32511, Egypt; 3 Department of Tanning Materials and Leather Technology, National Research Centre, Dokki, Giza 12611, Egypt; 4 Department of Botany and Microbiology, Faculty of Science, Menoufia University, Shebin El-Kom 32511, Egypt; 5 Chemistry Department, Faculty of Science, Tanta University, Tanta 31511, Egypt

**Keywords:** isatin, aminoquinoline, conjugates, bactericidal activity, antibiofilm, TEM

## Abstract

Isatin–quinoline conjugates **10a**–**f** and **11a**–**f** were assembled by the reaction of N-(bromobutyl) isatin derivatives **3a**, **b** with aminoquinolines **6a**–**c** and their corresponding hydrazinyl **9a**–**c** in good yields. The structures of the resulting conjugates were established by spectroscopic tools and showed data consistent with the proposed structures. In vitro antibacterial activity against different bacterial strains was evaluated. All tested conjugates showed significant biocidal activity with lower MIC than the first line drugs chloramphenicol and ampicillin. Conjugates **10a**, **10b** and **10f** displayed the most potent activity against all clinical isolates. The antibiofilm activity for all tested conjugates was screened against the reference drug vancomycin using the *MRSA* strain. The results revealed that all conjugates had an inhibitory activity against biofilm formation and conjugate. Conjugate **11a** showed 83.60% inhibition at 10 mg/mL. In addition, TEM studies were used to prove the mechanism of antibacterial action of conjugates **10a** and **11a** against (*MRSA*). Modeling procedures were performed on **10a**–**f** and **11a**–**f** and interestingly the results were nearly consistent with the biological activities. In addition, in silico pharmacokinetic evaluation was performed and revealed that the synthesized compounds **10a**–**f** and **11a**–**f** were considered drug-like molecules with promising bioavailability and high GI absorption. The results confirmed that the title compounds caused the disruption of bacterial cell membranes and could be used as potential leads for the further development and optimization of antibacterial agents.

## 1. Introduction

Bacterial infections comprise most hospital- and community-acquired infections [1,2]. The emergence and wide spread of drug-resistant pathogens has exacerbated the situation [3]. Finding new antibacterial drugs is one of the challenges that researchers face in overcoming the emergence of antibiotic resistance to various bacterial strains [4]. The misuse and overuse of antibiotics, as well as mutation owing to environmental changes and the gene transfer of bacteria, are the leading causes of antibiotic resistance [5]. From this perspective, searching for new natural or synthetic-based drugs to keep up with the rapid emergence of bacterial resistance is one of the main targets of medicinal chemistry. Nature contains various pharmaceutical compounds with potent biological activity [6,7]. Naturally occurring indole, quinoline and indoloquinoline heterocyclic cores are particularly important due to their diverse biological activities, which inspire many researchers to synthesize series of hybrid systems with different spacers [8,9,10,11]. Natural and synthetic isatin scaffolds have received much attention from many researchers due to their wide pharmaceutical application as antimicrobial, antioxidant, anticancer and antimalarial agents [12,13,14,15]. On the other hand, quinoline’s core structure represents a promising surrogate in drug design and development. This N-containing heterocyclic skeleton is incorporated in many natural and synthetic biologically active molecules [16,17,18,19,20,21,22] such as chloroquine and its analogues, which are well known drugs used to treat malaria [23,24]. Encouraged by the aforementioned facts, it is anticipated that by combining two intrinsically biologically active motifs such as isatin and quinoline moiety a synergistic effect will be obtained for the resulting surrogate’s structure when compared with each moiety independently [25,26,27].

**Figure 1:**
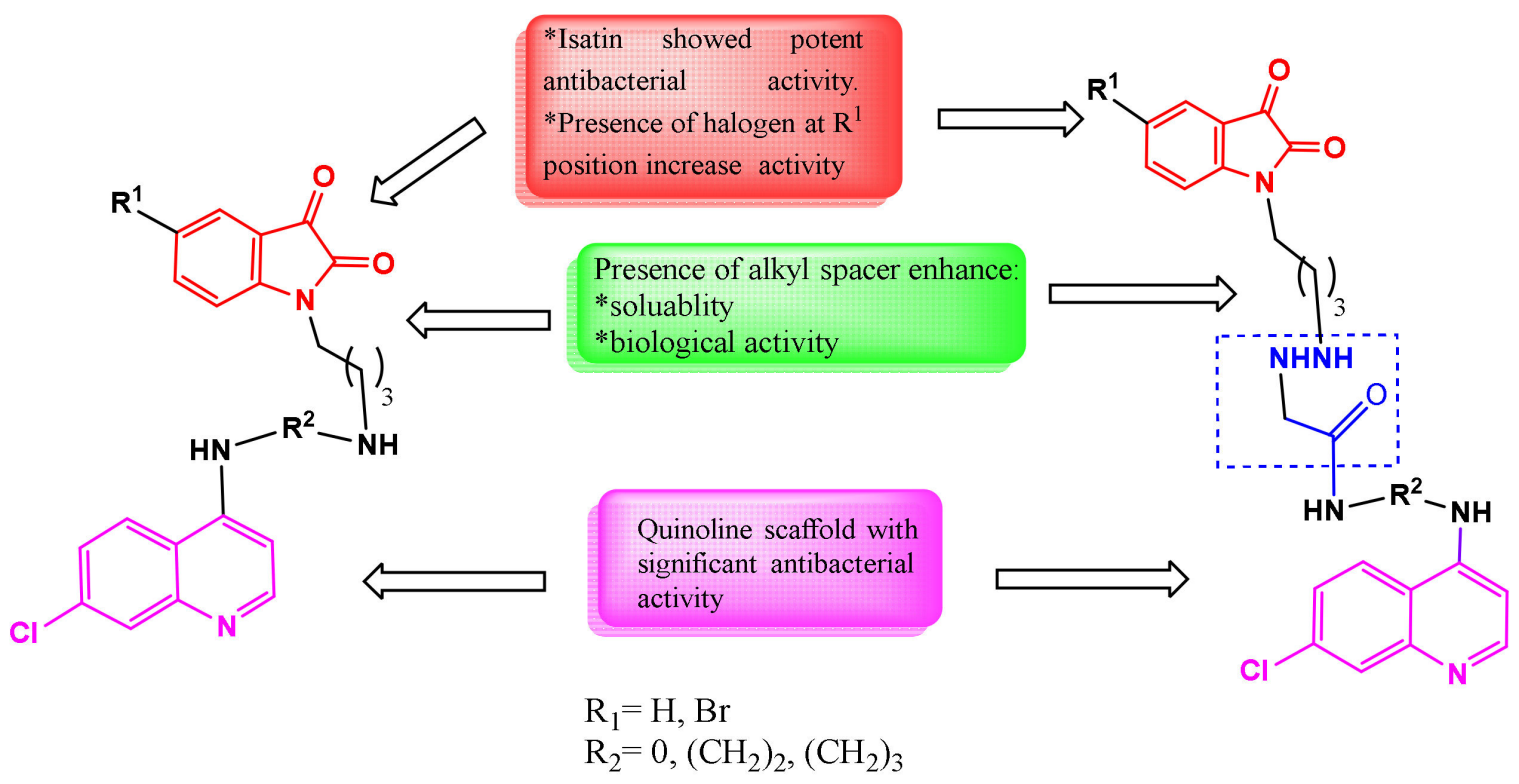
Design strategy for target conjugates containing isatin–quinoline motifs.

Previous studies have reviewed the biological importance of isatin–quinoline hybrids and illustrated the potent activity of these conjugates [28,29]. Moreover, the presence of linkers between isatin and quinoline have shown a significant role in enhancing the biological activities of these conjugates [30,31]. Therefore, many researchers seek to enhance and optimize the biological activity of isatin–quinoline conjugates. From the above-mentioned information, the present research work emphasizes the synthesis and biological evaluation of some novel isatin–quinoline conjugates with the presence of alkyl spacers aiming to reach the best activity for the synthesized conjugates, Figure 1.

## 2. Results and Discussion

### 2.1. Chemistry

**Scheme 1:**
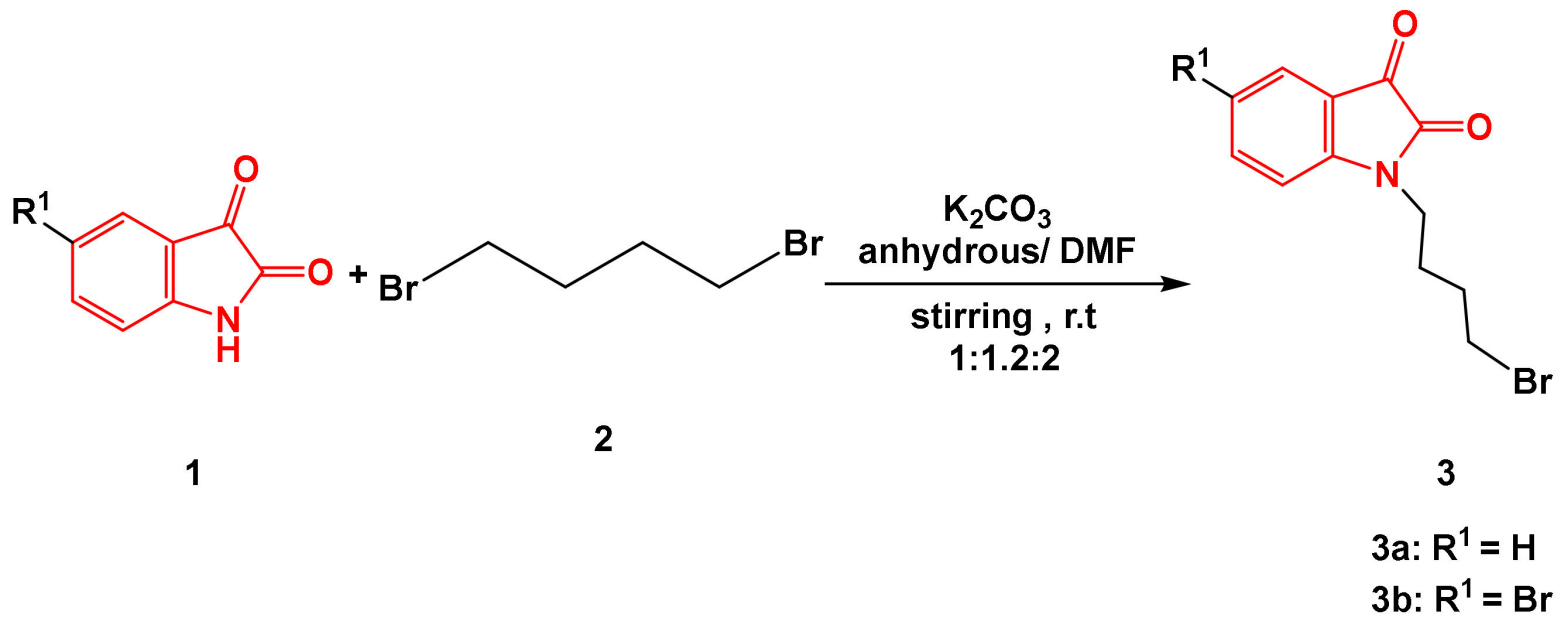
Synthesis of *N*-bromobutyl isatin derivative **3**.

The preparation of N-(bromobutyl) isatin derivatives **3a**,**b** was accomplished in good yields according to the method in the literature [32,33], as depicted in Scheme 1. 

**Scheme 2:**
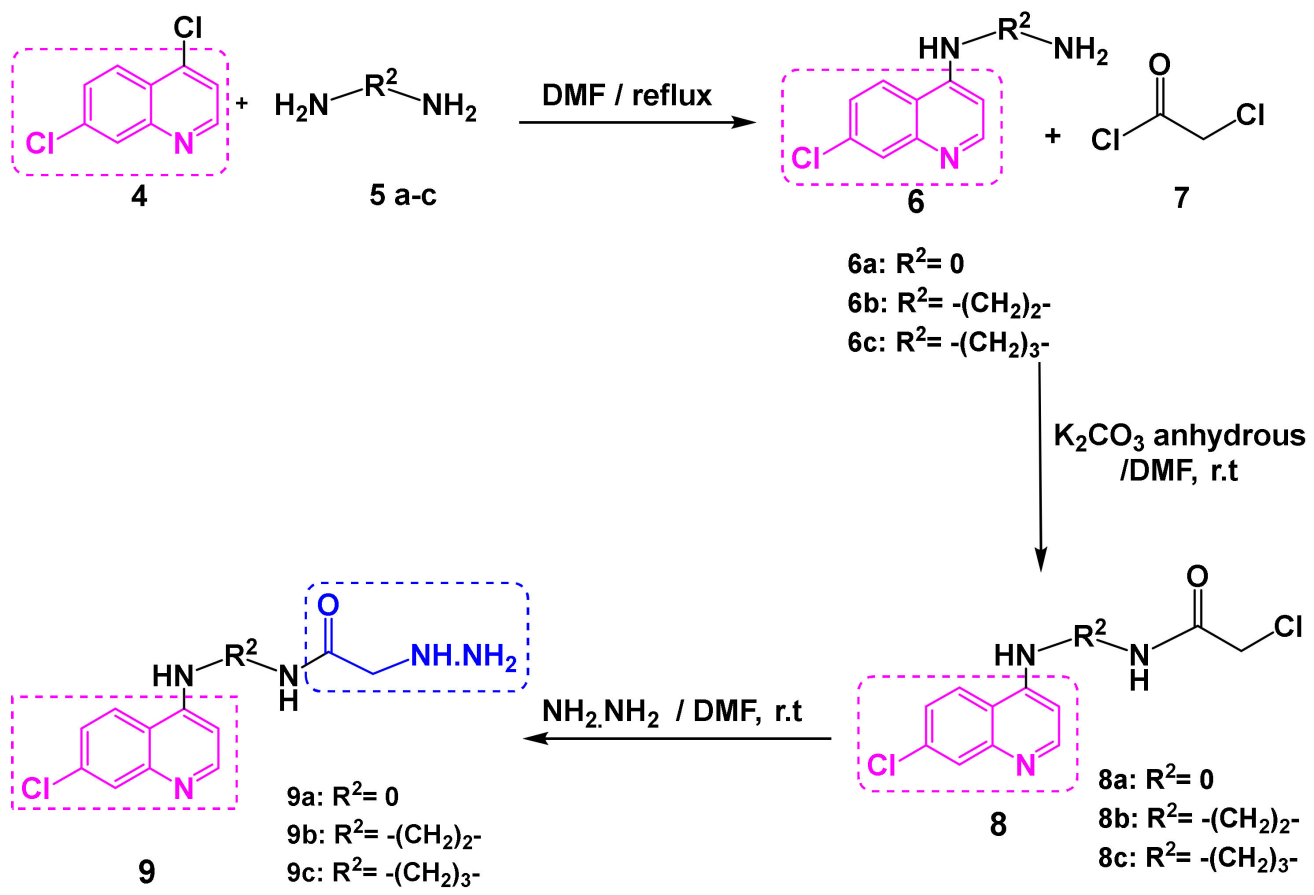
Synthesis of hydrazide derivatives **9a**–**c**.

Moreover, aminoquinolines **6a**–**c** and their corresponding hydrazinyl **9a**–**c** were synthesized in good yields starting from the reaction of 4,7-dichloroquinoline **4** with excess hydrazine or aliphatic diamine **5a**–**c** in DMF under reflux condition according to the method used in the literature [30]. Further reaction of **6a**–**c** with chloroacetyl chloride **7** in DMF in the presence of anhydrous potassium carbonate afforded the corresponding intermediates **8a**–**c** in good yields according to the reported method [32]. Moreover, the reaction of **8a**–**c** with excess hydrazine in DMF at room temperature yielded the corresponding hydrazinyl derivatives **9a**–**c** in good yields as illustrated in Scheme 2.

**Scheme 3:**
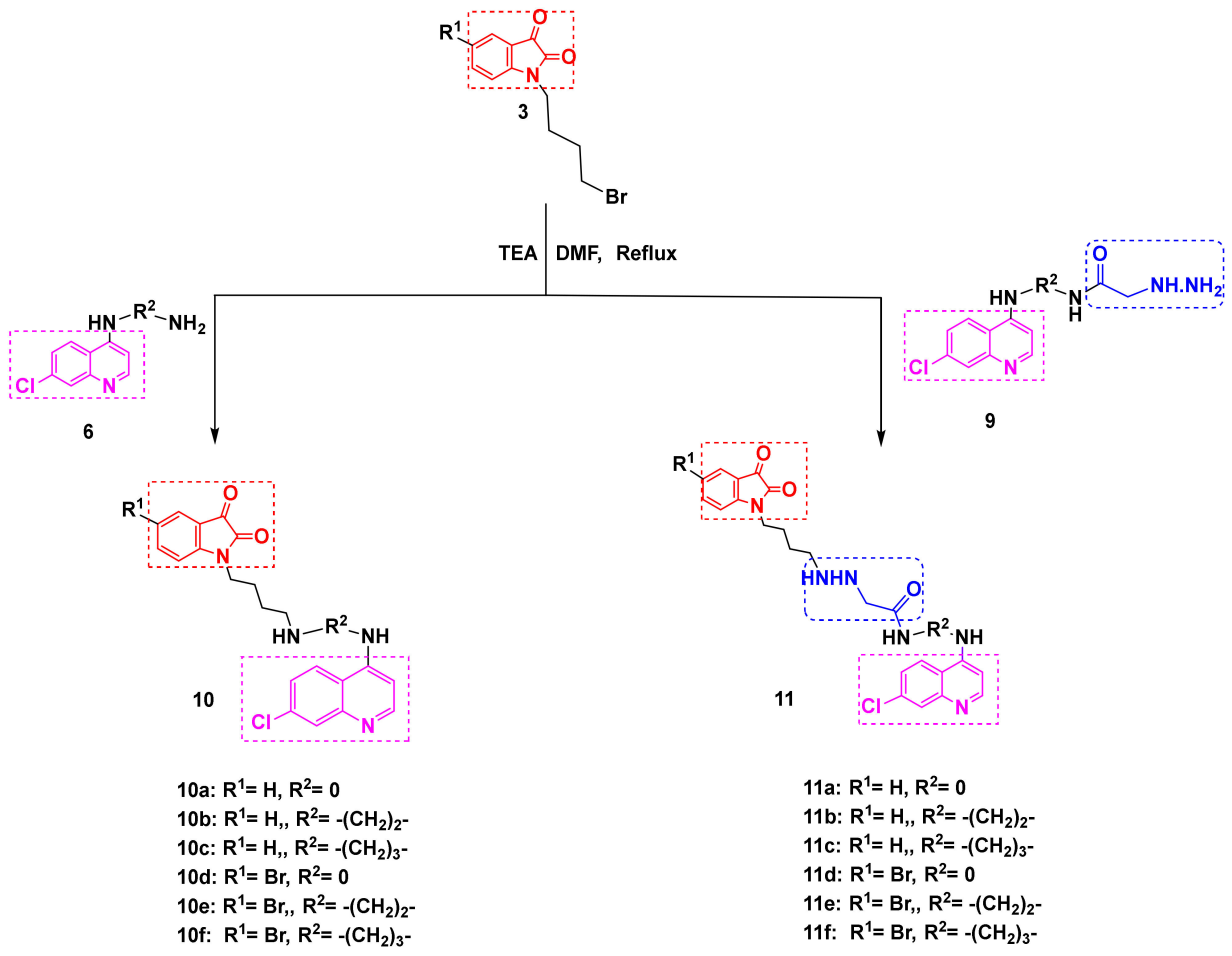
Synthesis of isatin—quinoline conjugates of type **10a**–**f** and **11a**–**f**.

Finally, the new conjugates **10a**–**f** and **11a**–**f** bearing isatin and quinoline motifs were achieved in good yields by the reaction of **3a**,**b** with **6a**–**c** or **9a**–**c** in the presence of triethyl amine in DMF under reflux condition for 4h until the consumption of the reactants as monitored by TLC chromatography as depicted in Scheme 3.

For the structural elucidation of **10a**–**f**, the FT-IR showed υ_(NH)_ absorption ranging from 3397 to 3312 cm^−1^. In addition, for υ _(>N-C=O)_ of C-2 the isatin core appeared from 1675 to 1653 cm^−1^ and 1731 to 1703 cm^−1^for υ _(>C=O)_ of C-3 in the isatin core. The ^1^H-NMR spectra performed in (CDCl_3_) confirmed the formation of isatin—quinoline conjugates by a noticeable increase in the number of chemical shifts in the aliphatic and aromatic ranges, the appearance of the (N-CH_2_) signal ranging from δ: 3.79 to 4.28 ppm and the characteristic peak quinoline (CH=N) was reported by signals at a range from δ: 8.54 to 8.82 ppm as singlet. Moreover, the ^13^C-NMR spectra performed in (CDCl_3_) showed an aliphatic chain ranging from δ: 24.07 to 53.44 ppm. Furthermore, the quinoline (CH=N) showed signals ranging from δ: 150.23 to 158.24 ppm and the (>N-C=O) group of the isatin core recorded for position C-2 from δ: 151.02 to 162.55 ppm and C-3 from δ: 181.95 to 184.55 ppm as illustrated in Table 1.

Meanwhile, the structure characterization of isatin—quinoline conjugates of type **11a**–**f** confirmed the formation of target conjugates as the FT-IR absorption bands showed the presence of υ _(NH)_ from 3405 to 3293 cm^−1^ and the υ _(>C=O)_ of C-2 was reported as ranging from 1657 to 1653 cm^−1^, and for the other υ _(>C=O)_ of C-3 of the isatin core from 1731 to 1705 cm^−1^. Furthermore, the ^1^H-NMR spectra showed the (*N*-CH_2_) for **11a**–**f** ranging from δ: 3.79 to 4.31 ppm and for quinoline (CH=N) showed at a range from δ: 8.54 to 8.82 ppm. In ^13^C-NMR, for the (CH=N) signals were reported at a range from δ: 149.08 to 150.55 ppm and the (>N-C=O) group of the isatin core recorded for position C-2 from δ: 150.71 to 162.62 ppm, while the (>C=O) group-bearing hydrazine group appeared from δ: 170.19 to 178.83 ppm and C-3 from δ: 181.96 to 184.53 ppm as shown in Table 1.

### 2.2. In Vitro Antibacterial Screening

The antibacterial properties of conjugates **10a**–**f** and **11a**–**f** at a concentration of 40 mg/mL were assessed against clinical isolates. In this study, the most resistant bacterial strains such *MRSA, Streptococcus mutans, Klebsiella pneumoniae* and *Serratia marcescens* were used against first line drugs ampicilin and chloramphnicol as a reference. The results revealed that all conjugates had bactericidal activity higher than the reference drugs (ampicilin and chloramphnicol) as well as the mother cores of synthesized conjugates (isatin derivatives and 4.7 dichloroquinoline).

#### 2.2.1. Antibacterial Screening of Conjugates **10a**–**f** and **11a**–**f** against Methicillin-Resistant *Staphylococcus aureus* (MRSA)

As stated in Table 2 and Figure S1, all conjugates were screened against *MRSA* clinical isolate *MRSA* ATCC 43300 and displayed higher activity than the controls. It is noticeable that **11a** had the maximum zone of inhibition against *MRSA* with inhibition zone (47.33 ± 0.60 mm), followed by conjugate **11c** with inhibition zone (30.72 ± 1.00 mm), while the other conjugates showed variable activity ranging from 20 to 25 mm and the ones with least effect were **11e** and **11f** with zones of inhibition 18.31 ± 0.60 and 16.34 ± 0.60 mm, respectively. In addition, the MIC values of **10** and **11** ranged from 0.006 mg/mL to 2.5 mg/mL and the MBC values ranged from 0.05 mg/mL to 5 mg/mL for **10a**–**f** and **11a**–**f**.

#### 2.2.2. Antibacterial Screening of Conjugates **10a**–**f** and **11a**–**f** against Streptococcus Mutans

As reported in Table 3 and Figure S2, all the compounds of **10a**–**f** and **11a**–**f** were screened against *Streptococcus mutans*. Compound **11a** showed the highest zone of inhibition against *Streptococcus mutans* (46.3 ± 1mm), others showed variable activity ranging from 18 to 24 mm and the least effective one was **11f** (14.3 ± 0.6 mm). As shown in Table 2, we compared the *Streptococcus mutans* clinical isolate and *Streptococcus mutans* ATCC 35668. The MIC values ranged from 0.0002 mg/mL to 2.5 mg/mL and MBC values ranged from 0.0004 mg/mL to 5 mg/mL for **10a**–**f** and **11a**–**f**.

#### 2.2.3. Antibacterial Screening of Conjugates **10a**–**f** and **11a**–**f** against *Klebsiella pneumoniae*

As shown in Table 4 and Figure S3, conjugate **11a** had the best zone of inhibition against *Klebsiella pneumoniae* (41.6 ± 0.6mm), while the other conjugates showed variable activity ranging from a 19 to 25 mm zone of inhibition. On the other hand, the least potent analogues were **10b** and **11f** with ZOI (17 ± 1, 16 ± 1, respectively). As shown in Table 4, we compared the *Klebsiella pneumoniae* clinical isolate and *Klebsiella pneumoniae* ATCC 700603. The MIC values ranged from 0.0008 mg/mL to 2.5 mg/mL and the MBC values ranged from 0.006 mg/mL to 5 mg/mL for **10a**–**f** and **11a**–**f**.

#### 2.2.4. Antibacterial screening of conjugates **10a**–**f** and **11a**–**f** against Serratia marcescens

As shown in Table 5 and Figure S4, **11a** had the maximum zone of inhibition against *Serratia marcescens* (43 ± 1mm), followed by **11c** (32.3 ± 0.6 mm) and other conjugates varying between 21.7–28 mm; the least effective ones were **11f**, **11b** and **11e** (10.7 ± 0.6 mm, 15.3 ± 0.6 mm and 17.3 ± 0.6 mm, respectively). All the conjugates were higher than the antibiotics that were used as positive control except **11f**. As shown in Table 4, we compared the *Serratia marcescens* clinical isolate and *Serratia marcescens* ATCC13880. The MIC values ranged from 0.0004 mg/mL to 80 mg/mL and the MBC values ranged from 0.0008 mg/mL to 160 mg/mL for **10a**–**f** and **11a**–**f**.

### 2.3. Time-Kill Assay

**Figure 2:**
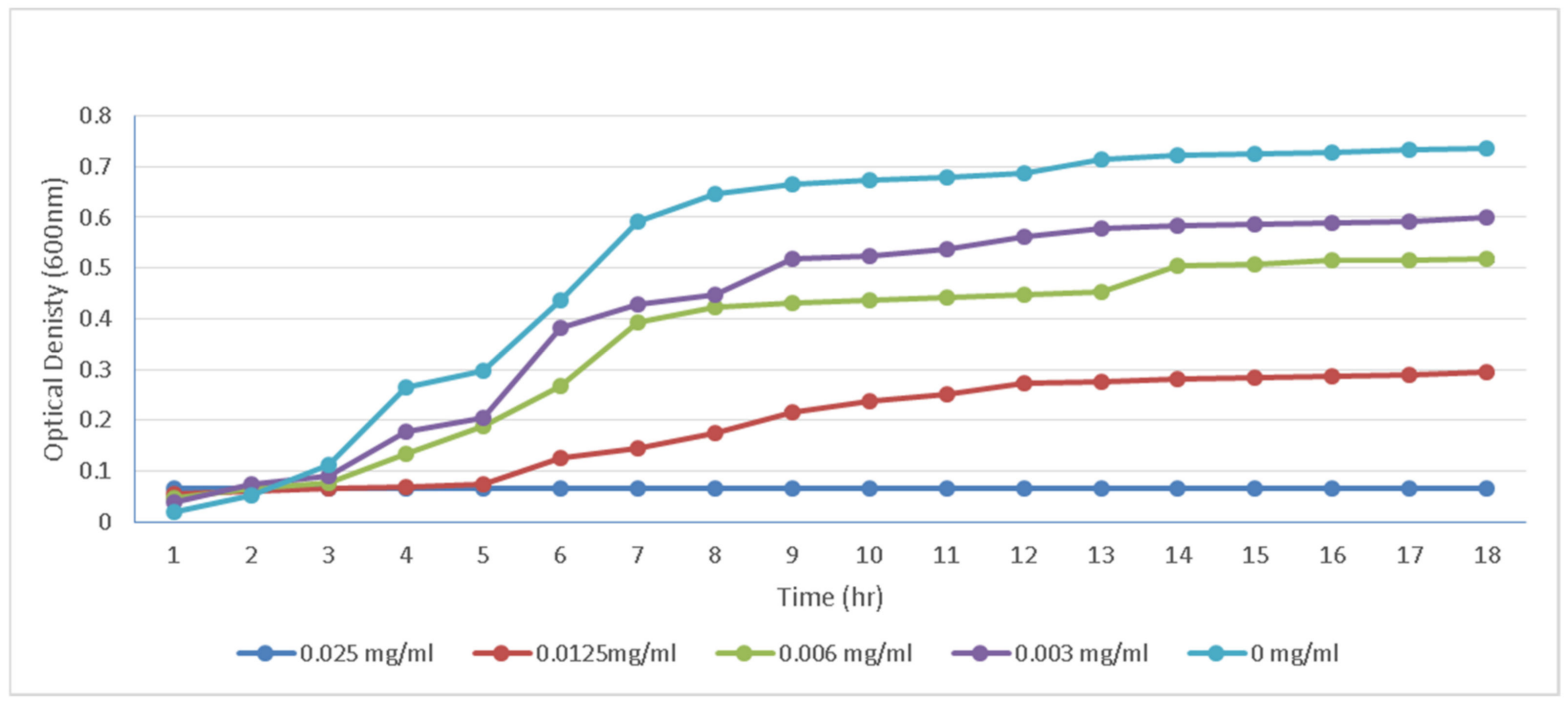
Time-kill plot for *MRSA* in the presence of **10a**.

**Figure 3:**
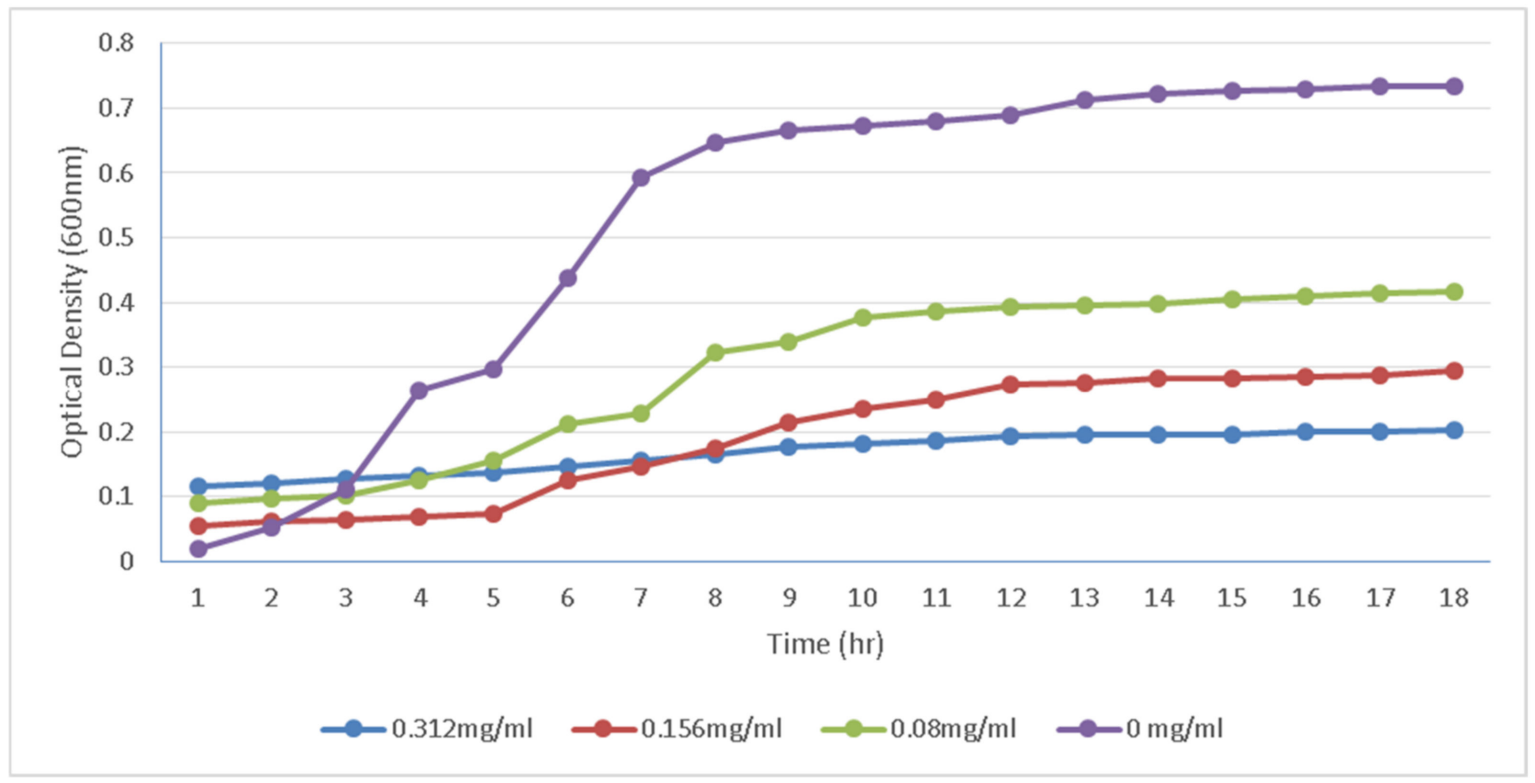
Time-kill plot for *MRSA* in the presence of **11a**.

All conjugates had a MBC/MIC ratio ≤ 4, (bactericidal), as illustrated in Table 6. Therefore, they were tested for the time-kill assay [34,35]. The results obtained for the time-kill study of *MRSA* are shown in Figure 2 and Figure 3 for **10a** and **11a,** respectively, as they are the best conjugates in the two series. The MBC values for **10a** and **11a** were consistent with the cidal concentration as displayed in the growth curve of this time-kill study. This study supported that the antibacterial activity is dose dependent. The data in Table 6 revealed that the activity depends on structure variations as seen from the value of the MIC among the tested conjugates. The lowest MIC values (0.006 and 0.025 mg/mL) were in **10a**, **10c**, **10d**, **10e** and **10f** against *MRSA*, and **10b** had a MIC of (0.0125 mg/mL); also, **11a** and **11c** had a MIC value of (0.156 mg/mL) and finally the highest MIC values were in **11b**, **11d**, **11e** and **11f**. The difference in MIC of conjugates **10** and **11** was due to the variation in their chemical structure. In general, conjugates of **10** had lower MIC values than conjugates of **11**, as they could affect multiple target sites against bacterial cells.

### 2.4. Assessment of Anti-Biofilm Assay

This method allowed the determination of the anti-biofilm property of tested conjugates versus vancomycin using the 96-well plate. The biomass of bacterial cells was quantitatively analyzed on the microplate reader at an absorbance of 570 nm, showing significant reduction of biofilm when an increasing concentration of the tested conjugates was used when compared to the control (TSB only). The results showed that tested conjugates at 10 mg/mL exhibited the most significant anti-biofilm activity, ranging from 79.4 ± 0.22 to 62.9 ± 0.07% in conjugate **10**, while ranging in conjugate **11** from 83.6 ± 0.11 to 50.2 ± 0.11%. Meanwhile, the anti-biofilm activity of the positive control vancomycin was measured at 43 ± 0.17% at the higher concentration of 100 mg/mL [36] as reported in Table 7 and Table 8 and Figures S5–S7.

### 2.5. Transmission Electron Microscopy (TEM) Assay 

**Figure 4:**
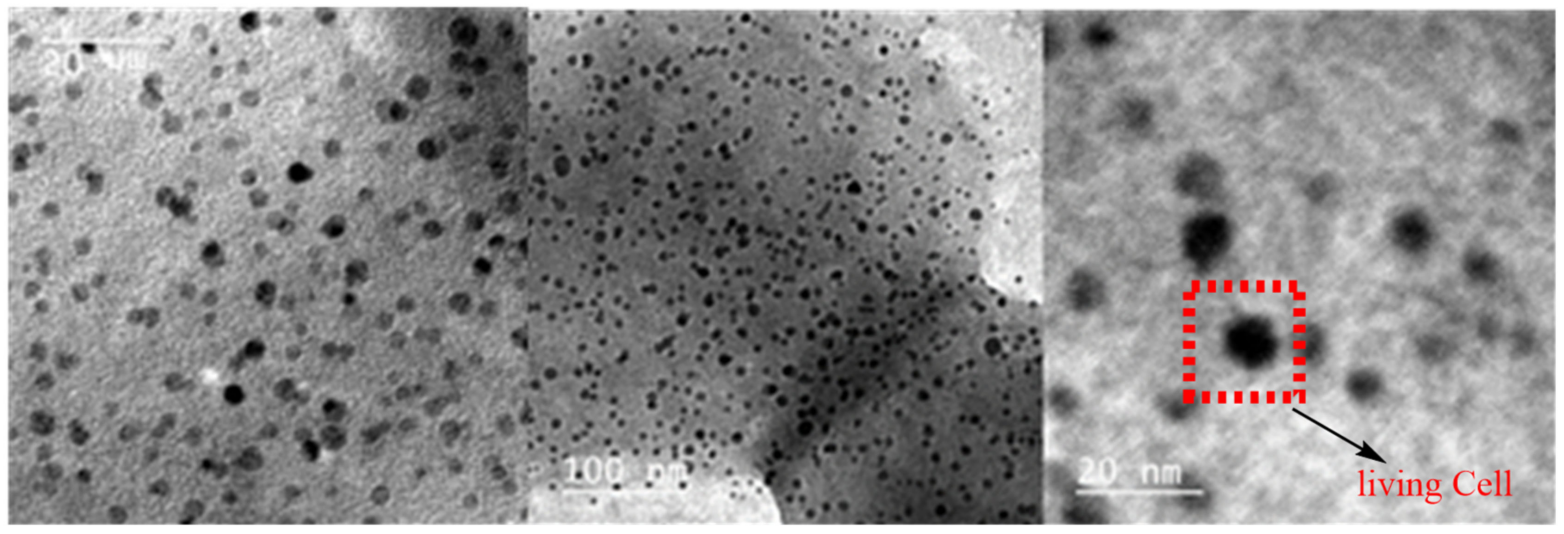
TEM micrographs of untreated methicillin-resistant *Staphylococcus aureus* (*MRSA*).

**Figure 5:**
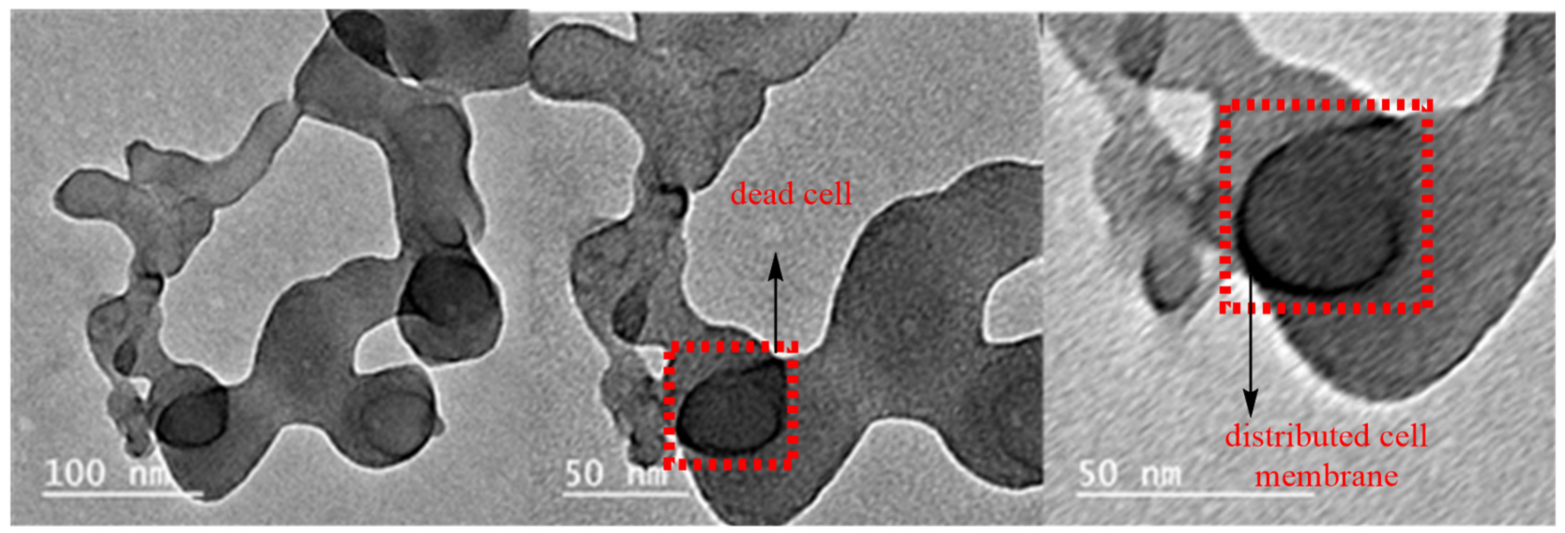
TEM micrographs of methicillin-resistant *Staphylococcus aureus* (*MRSA*) treated with **10a** showing incomplete cell wall destruction, cell death.

**Figure 6:**
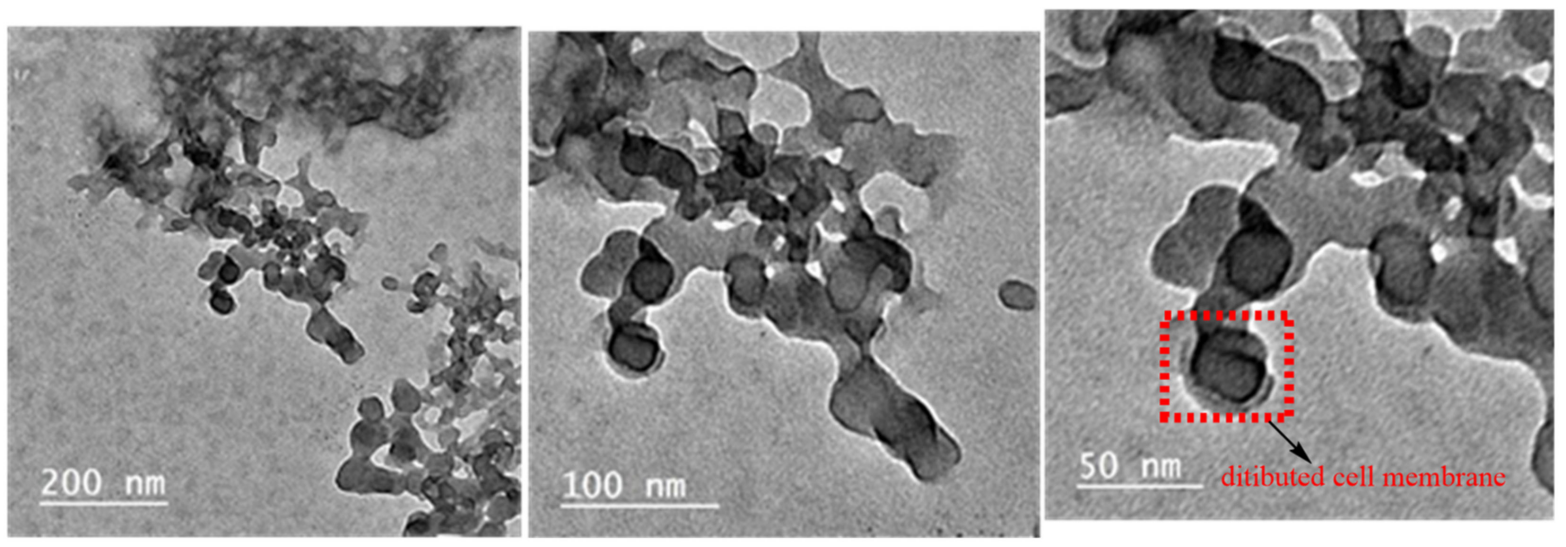
TEM micrographs of methicillin-resistant *Staphylococcus aureus* (*MRSA*) treated with **11a** showing incomplete cell wall destruction, cell death.

The structural appearance of untreated *MRSA* as depicted in Figure 4 with treated **10a** and **11a** as shown in Figure 5 and Figure 6, respectively, was analyzed using TEM. The figures of treated *MRSA* show an altered *MRSA* cell membrane and dead bacterial cells after exposure to conjugates. The figures also show vacuoles created in the cytoplasm. These micrographs demonstrate that the compounds disrupt the *MRSA* bacterial cell wall, resulting in cell death; these effects were amplified and can be described as bactericidal. The figure also depicts increased cell death because of fewer cells, which is consistent with the observations made with untreated *MRSA* in Figure 4.

### 2.6. Molecular Docking

Molecular docking studies were performed on the prepared compounds **10a**–**f** and **11a**–**f**. The target enzyme crystal structure bound to its co-crystallized ligand was downloaded from Protein Data Bank (PDB:4DKI) [37]. An in silico screening study of the prepared structures was carried out at the transpeptidase enzyme active site in comparison with the co-crystalized ligand.

**Figure 7:**
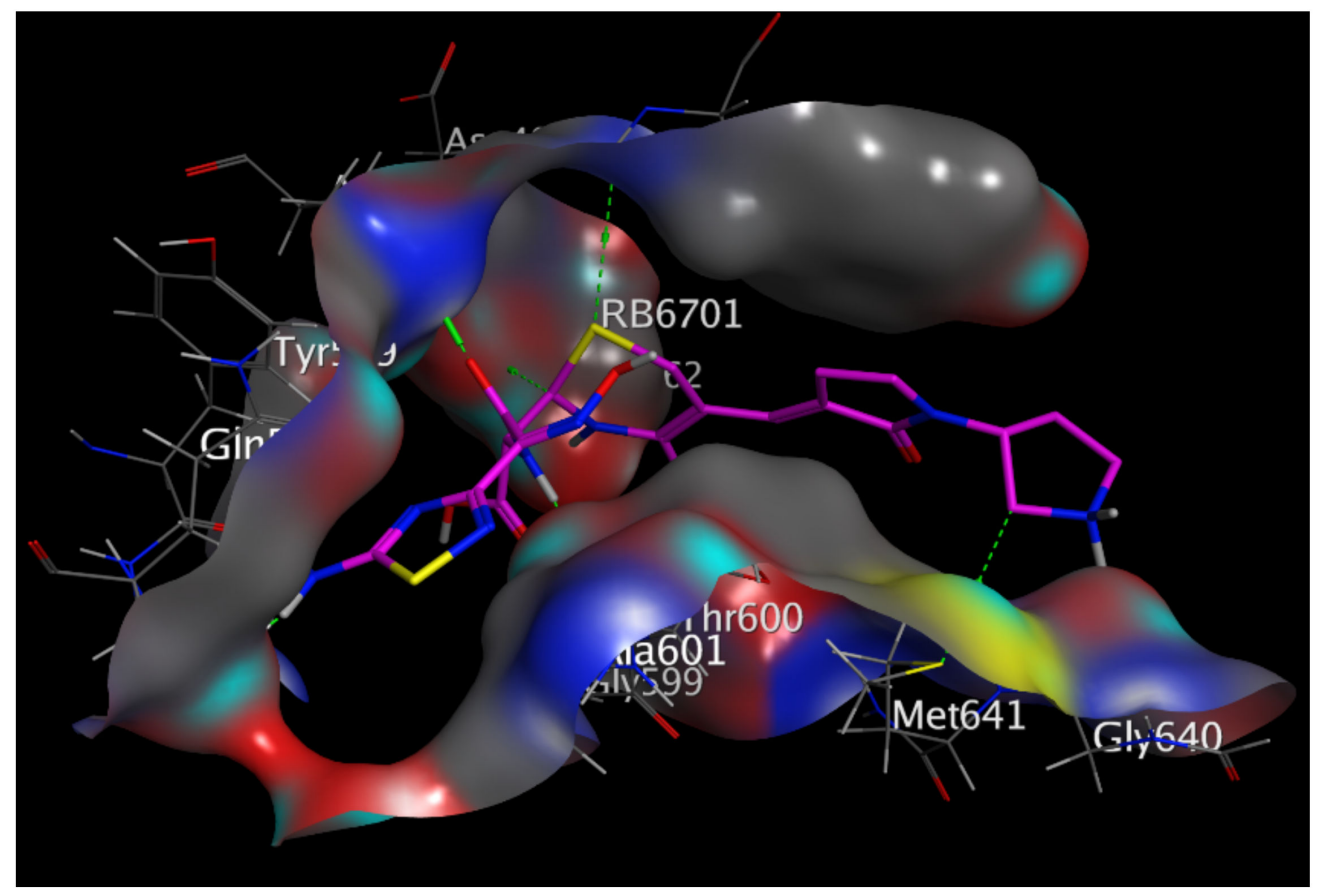
Co-crystallized ligand (RB6) represented as pink sticks re-docked in the receptor active site.

**Figure 8:**
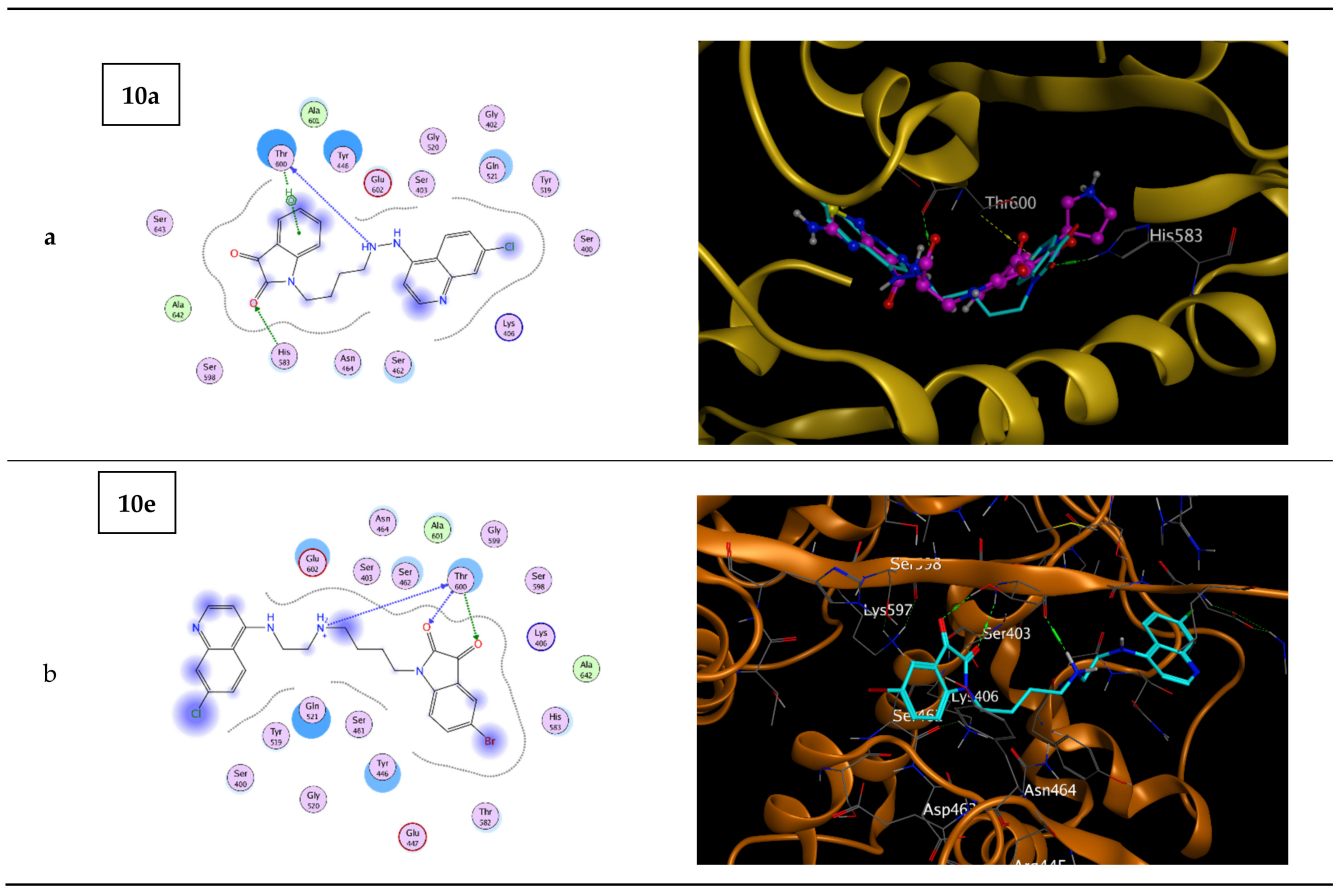

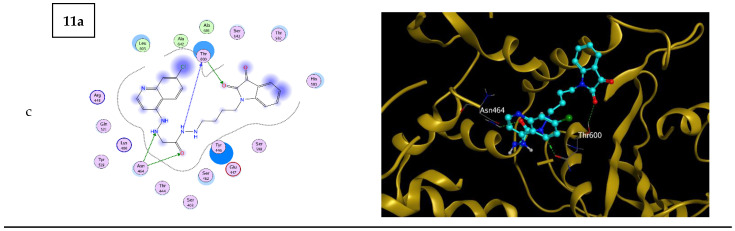
Compound **10a**, **10e** and **11a** interactions inside the active site with H-bonds in green and H-Pi interaction in yellow. (**a**) **10a** (in cyan sticks overlapped with the co-crystallized ligand in magenta ball and stick), (**b**) **10e** (in cyan sticks), (**c**) **11a** (in cyan ball and stick).

A redocking step was performed for validation of results, Figure 7. Docking results of the synthesized compounds, including binding affinity score and root mean square deviation RMSD in addition to the ligand interactions with the active site residues, either hydrogen bonding or hydrophobic interactions, are tabulated below, Table 9, Figure 8. The receptor residues that were mainly involved in most interactions between the synthesized ligands and the active site were “THR 600, HIS583, GLN521, SER462 and TYR446”, Table 9.

### 2.7. Structure Activity Relationships Study (SARs)

From the previous biological study for the synthesized conjugates, **10** and **11** against Gram-positive and Gram-negative bacteria with variable concentration were all shown to have significant activity compared to the reference drugs ampicillin and chloramphenicol as well as higher activity than isatin and quinoline scaffolds, which affirmed the synergistic effect of these conjugates. 

For conjugates of type **10,** it was noticeable that **10b** showed the best activity against *MRSA* where the two-carbon spacer between quinoline and isatin moieties is attached; the same activity with **10e** was seen with C-5 of the isatin core bearing the bromine atom with the same two-carbon spacer. On the other hand, conjugate **10a** showed the best activity against *Streptococcus mutans* and *Klebsiella pneumoniae* compared to the other analogues of **10**. It is worthy of note that **10a** containing a hydrazine spacer has higher activity than **10d** having the same skeleton but containing the bromo atom at C-5 of the isatin core. Meanwhile, conjugates **10c** and **10f**, which have a three-carbon spacer with or without a bromo atom at C-5 of the isatin core, respectively, have more significant activity than the other conjugates of type **10** against *Serratia marcescens*. For the series of conjugates **11**, it is noticeable that **11a** has significant activity against all Gram-positive and Gram-negative bacteria compared to the other conjugates of type **11**. Comparing the conjugates of **10** and **11** with each other, it is worthy of note that **11a,** having Acyl-methyl hydrazinyl without the carbon spacer, is effective against all tested bacteria stains compared to **10a** containing the hydrazine spacer.

### 2.8. In silico Pharmacokinetic Assessment

The assessment of pharmacokinestic properties was performed on the prepared compounds **10a**–**f** and **11a**–**f**, using both Molsoft and Swiss ADME [38]. In reference to Lipinski’s rule of five, they all had no Lipinski’s rule violations except in the molecular weight of compounds **10e**, **10f** and **11c**–**f**, which exceeded 500. The lipophililicity of the tested compounds recorded ilog *p* < 5, which indicated their strong tolerance by cell membranes [39]. Topological polar surface area (TPSA) values ranged from 74 to 115. The number of hydrogen bond donors (HBD) was either two or four, and the number of hydrogen bond acceptors (HBA) ranged from four to six acceptors. Interestingly, all compounds showed high GI absorption ability. Moreover, the blood brain barrier (BBB) score was between zero and six as referenced [40], recording a minimum value of 2.25 and a maximum of 4.01, Table 10. Analyzing the drug likeness of the compounds, good drug-like candidates are considered to express positive drug-likeness scores [41], and as illustrated below in Table 10, positive values ranging from 0.25 to 0.66 were recorded by all the investigated compounds, among which compound **11a** was the most promising, recording a score of 0.66. All the investigated compounds showed good bioavailability scores and can be considered promising “drug-like” molecules, Table 10.

## 3. Materials and Methods

### 3.1. General

All NMR analyses with the Bruker magnet system 400’54 Ascend/R (USA) 400 MHz and 100 MHz for ^1^H-NMR and ^13^C-NMR, respectively, as well as FT-IR spectroscopy, were performed with Alpha-Bruker ATR mode (USA) at Zagazig University. The chemical shift of CDCl_3_ was recorded at 7.26 ppm. Mass spectrum was performed on Direct Inlet part to mass analyzer in GCMS model with ISQ single quadrupole thermoscientific Electron Impact mode (UK) at the Regional Center for Mycology and Biotechnology, Al-Azhar University. Melting points (m.p.) were determined using the Stuart scientific melting point apparatus and are uncorrected. The in vitro antibacterial screening was executed at the Department of Botany and Microbiology, Faculty of Science, Menoufia University. Without further purification, the solvents were used as received. Commercially available starting materials as isatin derivatives **1a**,**b,** free diamines **5a**–**c** and 4.7-dichloro quinoline **4** were purchased from Acros and Sigma Aldrich, Germany. Synthetic starting materials such as N-(bromobutyl) isatin derivatives **3a**–**c**, aminoquinoline derivatives **6a**–**c** and intermediates **8a**–**c** were synthesized as illustrated [32,33,42,43]. Mueller Hilton broth (Becton Dickinson, Sparks, MD, USA), nutrient broth, Tryptic soy broth (TSB) and nutrient agar were purchased from (Bacto), DMSO was purchased from R&M Marketing, Essex, UK. Iodonitrotetrazolium chloride (INT), as well as the first line antibiotics chloramphenicol, ampicillin and vancomycin hydrochloride were purchased from Sigma Aldrich, Germany. Other materials included the 96-wells microplate reader, 96-wells microtiter, 15 mL centrifuge tubes, which contained glucose and stimulate biofilm formation especially with Methicillin-resistant *Staphylococcus aureus*, Aqueous crystal violet (0.1% *w*/*v*), Glacial acetic acid (30% *v*/*v*), 1 × Phosphate buffer saline, pipette, as the positive control in antibiofilm test.

#### 3.1.1. Bacterial Strains

Clinical bacterial isolates were obtained from the National Research Center. Four different microbial (ATCC reference) cultures and four clinical isolates were used in all of the experiments.

#### 3.1.2. Reference Strains

Methicillin-resistant *Staphylococcus aureus*, *MRSA* (ATCC 43300), *Streptococcus mutans* ATCC 35668, *Klebsiella pneumoniae* ATCC 700603 and *Serratia marcescens* ATCC13880.

#### 3.1.3. Clinical Isolates

*Staphylococcus aureus*, MRSA, *Streptococcus mutans, Klebsiella pneumonia* and *Serratia marcescens.*

### 3.2. Chemistry

#### 3.2.1. General Procedure for Synthesis of 4-Bisamino Quinoline Hydrazine Derivatives **9**

To (1 mmol) of **8a**–**c** was added excess amount (3 mmol) of hydrazine, with stirring for 3h at room temperature (25 °C) in presence of (1 mL) of DMF until the consumption of the reactants, monitoring throughout by TLC. After the completion of the reaction, the reaction mixture was poured into ice water. The obtained products **9a**–**c** were filtered, washed off and recrystallized from methanol.

N-(6-chloronaphthalen-1-yl)-2-hydrazineylacetohydrazide (**9a**)

Pale yellow solid, yield (0.42 g, 71%), m.p = 160–162 °C, FT–IR (KBr) cm^−1^ υ: 3277 (NH), 3123 (NH_2_), 2940 (CH), 1725 (C=O), 1628 (C=C_Ar_), 1578 (C=N), 812 (C-Cl). ^1^H NMR (CDCl_3_, 400 MHz) ppm δ: 3.77 (s, 2H, CH_2_), 6.82–7.84 (m, 4H, CH_Ar_), 8.38 (br.m, 1H, CH=N_Ar_), 8.52 (br.m, 2H, NH_2_), 8.95 (br.m, 2H, 2NH). ^13^C–NMR (CDCl_3_, 100 MHz) ppm δ: 62.88, 111.85, 116.86, 118,70, 119.36, 121.33, 138.64, 149.11, 157.75, 178.31. EI–MS *m*/*z* (C_12_H_13_ClN_4_O): calcd., 265; found: 265 [M]^+^, 267 [M+2H]^+^.

N-(2-((6-chloronaphthalen-1-yl)amino)ethyl)-2-hydrazineylacetamide (**9b**)

Pale yellow solid, yield (0.48 g, 72%), m.p = 171–173 **°**C, FT–IR (KBr) cm^−1^ υ: 3338 (NH), 3179 (NH_2_), 2939 (CH), 1735 (C=O), 1632 (C=C_Ar_), 1587 (C=N), 869 (C-Cl). ^1^H–NMR (CDCl_3_, 400 MHz) ppm δ: 3.16 (br.s, 2H, CH_2_), 3.38 (s, 2H, CH_2_), 3.78 (br.s, 2H, CH_2_), 7.36–8.18 (m, 4H, CH_Ar_), 8.77 (br.s, 1H, CH=N_Ar_) 8.78 (br.m, 2H, NH_2_), 8.91 (br.m, 2H, 2NH). ^13^C–NMR (CDCl_3_, 100 MHz) ppm δ: 39.52, 51.09, 53.52, 121.44, 125.65, 128.50, 128.84, 136.80, 140.75, 143.11, 149.08, 150.71, 175.15. EI–MS *m*/*z* (C_14_H_17_ClN_4_O): calcd., 293; found: 293 [M]^+^, 295 [M + 2H]^+^.

N-(3-((6-chloronaphthalen-1-yl)amino)propyl)-2-hydrazineylacetamide (**9c**)

Off-white solid, yield (0.46 g, 70%), m.p = 185–187 °C, FT–IR (KBr) cm^−1^ υ: 3411 (NH+NH_2_), 2940 (CH), 1727 (C=O), 1662 (C=C_Ar_), 1595 (C=N), 817 (C-Cl). ^1^H–NMR (CDCl_3_, 400 MHz) ppm δ: 1.89 (t, 2H, CH_2_, *J* = 8Hz), 3.48 (br.s, 4H, 2CH_2_), 3.63 (t, 2H,CH_2_, *J* = 8Hz), 4.16 (br.s, 2H, NH_2_), 7.29–8.28 (m, 4H, CH_Ar_), 8.45 (d, 1H, CH=N_Ar_), 8.73 (br.s 2H, 2NH). ^13^C–NMR (CDCl_3_, 100 MHz) ppm δ: 22.68, 29.87, 36.59, 55.34, 116.51, 119.34, 121.51, 130.52, 131.08, 131.68, 132.14, 142.42, 172.47. EI–MS *m*/*z* (C_15_H_19_ClN_4_O): calcd., 307; found: 307 [M]^+^, 309 [M+2H]^+^.

#### 3.2.2. General Procedure for Synthesis of Conjugates of **10** and **11**

To (1 mmol) **3a**,**b** equimolar ratio of aminoquinoline derivatives **6a**–**c** or **9a**–**c** (1 mmol) was added in presence of excessive amount (3mmol) of triethyl amine and 3 mL of DMF under reflux for 4h to afford the target conjugates **10a**–**f** and **11a**–**f** after the consumption of the reactants. The reaction mixture was poured into ice water then filtered off and recrystallized from methanol.

1-(4-(2-(7-chloroquinolin-4-yl)hydrazineyl)butyl)indoline-2,3-dione (**10a**)

Brown solid, yield (0.26 g, 64%), m.p = 205–207 °C, FT–IR (KBr) cm^−1^ υ: 3374 (NH), 2930 (CH), 1710 (C=O), 1675 (C=O), 1607 (C=C_Ar_), 1562 (C=N), 1146 (C-C), 745 (C-Cl). ^1^H–NMR (CDCl_3_, 400 MHz) ppm δ: 1.90 (br.m, 4H, 2CH_2_), 3.79 (br.m, 4H, CH_2_ + *N*-CH_2_), 6.89–8.12 (m, 8H, CH_Ar_), 8.54 (br.s, 1H, CH=N_Ar_), 13.22 (s, 1H, NH), 13.56 (s, 1H, NH). ^13^C–NMR (CDCl_3_, 100 MHz) ppm δ: 31.65, 36.95, 41.65, 99.76, 103.84, 117.85, 124.31, 125.33, 125.97, 126.34, 137.52, 150.23, 153.10, 167.35, 183.20. EI–MS *m*/*z* (C_21_H_19_ClN_4_O_2_): calcd., 394; found: 394 [M]^+^, 396 [M+2H]^+^.

1-(4-((2-((7-chloroquinolin-4-yl)amino)ethyl)amino)butyl)indoline-2,3-dione (**10b**)

Reddish-orange solid, yield (0.27 g, 71%), m.p = 215–217 °C, FT–IR (KBr) cm^−1^ υ: 3319 (NH), 2936 (CH), 1703 (C=O), 1665 (C=O), 1608 (C=C_Ar_), 1577 (C=N), 1224 (C-C), 748 (C-Cl). ^1^H–NMR (CDCl_3_, 400 MHz) ppm δ: 1.95 (br.m, 4H, 2CH_2_), 3.47 (br.m, 4H, 2CH_2_), 3.78 (br.m, 2H, CH_2_), 3.88 (br.m, 2H, *N*-CH_2_), 6.90–8.39 (m, 8H, CH_Ar_), 8.77(br.s, 1H, CH=N_Ar_), 13.80 (s, 1H, NH), 13.87 (s, 1H, NH). ^13^C–NMR (CDCl_3_, 100 MHz) ppm δ: 24.09, 25.90, 29.61, 34.79, 35.89, 44.81, 110.67, 117.37, 123.10, 124.42, 124.94, 126.87, 130.15, 136.24, 138.11, 142.79, 150.61, 158.17, 183.40. EI–MS *m*/*z* (C_23_H_23_ClN_4_O_2_): calcd., 422; found: 422 [M]^+^, 424 [M+2H]^+^.

1-(4-((3-((7-chloroquinolin-4-yl)amino)propyl)amino)butyl)indoline-2,3-dione (**10c**)

Pale yellow solid, yield (0.25 g, 73%), m.p = 232–234 °C, FT–IR (KBr) cm^−1^ υ: 3397 (NH), 2931 (CH), 1713 (C=O), 1654 (C=O), 1609 (C=C_Ar_), 1559 (C=N), 1231 (C-C), 750 (C-Cl). ^1^H–NMR (CDCl_3_, 400 MHz) ppm δ: 1.85 (br.m, 6H, 3CH_2_), 3.24 (m, 4H, 2CH_2_), 3.77 (m, 2H, CH_2_), 4.28 (br.m, 2H, *N*-CH_2_), 6.79–8.18 (m, 8H, CH_Ar_), 8.55 (br.s, 1H, CH=N_Ar_), 13.38 (s, 1H, NH), 13.72 (s, 1H, NH).^13^C–NMR (CDCl_3_, 100 MHz) ppm δ: 24.43, 29.69, 31.33, 36.47, 39.50, 49.80, 110.15,116.70, 119.18, 121.77, 123.93, 125.58, 127.10, 133.60, 138.57, 140.48, 155.49, 162.39 184.53. EI–MS *m*/*z* (C_24_H_25_ClN_4_O_2_): calcd., 436; found: 436[M]+, 438 [M+2]^+^.

5-bromo-1-(4-(2-(7-chloroquinolin-4-yl)hydrazineyl)butyl)indoline-2,3-dione (**10d**)

Brown solid, yield (0.35 g, 76%), m.p = 208–210 °C, FT–IR (KBr) cm^−1^ υ: 3360 (NH), 2933 (CH), 1732 (C=O), 1655 (C=O), 1606 (C=C_Ar_), 1567 (C=N), 1253 (C-C), 808 (C-Cl), 660 (C-Br). ^1^H–NMR (CDCl_3_, 400 MHz) ppm δ: 1.33 (br.m, 4H, 2CH_2_), 3.26 (br.m, 2H, CH_2_), 3.91 (br.m, 2H, *N*-CH_2_), 7.42–8.07 (m, 7H, CH_Ar_), 8.82 (br.s, 1H, CH=N_Ar_), 13.58 (s, 1H, NH), 13.71 (s, 1H, NH). ^13^C– NMR (CDCl_3_, 100 MHz) ppm δ: 29.68, 31.43, 36.47, 39.49, 116.75, 121.26, 121.38, 122.32, 123.92, 124.66, 125.55, 127.06, 127.65, 129.06, 129.30, 130.54, 131.08, 133.26, 138.58, 140.80, 158.24, 162.55, 183.29. EI–MS *m*/*z* (C_21_H_18_BrClN_4_O_2_): calcd., 473; found: 473[M]^+^, 475 [M+2]^+^.

5-bromo-1-(4-((2-((7-chloroquinolin-4-yl)amino)ethyl)amino)butyl)indoline-2,3-dione (**10e**)

Reddish-brown solid, yield (0.30 g, 72%), m.p = 212–214 °C, FT–IR (KBr) cm^−1^ υ: 3312 (NH), 2949 (CH), 1731 (C=O), 1657 (C=O), 1604 (C=C_Ar_), 1555 (C=N), 1269 (C-C), 815 (C-Cl), 577(C-Br). ^1^H–NMR (CDCl_3_, 400 MHz) ppm δ: 1.93 (br.m, 4H, 2CH_2_), 3.35 (br.m, 4H, 2CH2), 3.45 (br.m, 2H, CH_2_), 3.81 (br.m, 2H, *N*-CH_2_), 6.75–8.21 (m,7H, CH_Ar_), 8.81 (br.s, 1H, CH=N_Ar_), 13.17 (s, 1H, NH), 13.48 (s, 1H, NH). ^13^C–NMR (CDCl_3_, 100 MHz) ppm δ: 24.26, 35.07, 39.52, 53.44,111.82, 116.88, 121.45,125.05, 125.64, 128.38, 128.69, 128.76, 136.54, 140.78, 142.72,149.46, 151.02, 167.17, 181.95. EI–MS *m*/*z* (C_23_H_22_BrClN_4_O_2_): calcd., 501; found: 501 [M]^+^, 503 [M+2H]^+^.

5-bromo-1-(4-((3-((7-chloroquinolin-4-yl)amino)propyl)amino)butyl)indoline-2,3-dione (**10f**)

Violet solid, yield (0.30 g, 70%), m.p = 230–232 °C, FT–IR (KBr) cm^−1^ υ: 3343 (NH), 2939 (CH), 1705(C=O), 1653 (C=O), 1605(C=CAr), 1587(C=N), 1161(C-C), 808 (C-Cl), 585(C-Br). ^1^H–NMR (CDCl_3_, 400 MHz) ppm δ: 1.82 (br.m, 6H, 3CH_2_), 2.67 (m, 4H, 2CH_2_), 3.75 (t, 2H, CH_2_, *J* = 8 Hz), 3.89 (t, m, 2H, *N*-CH_2_, *J* = 8 Hz), 7.06–8.41 (m, 7H, CH_Ar_), 8.65 (d, 1H, CH=N_Ar_, *J* = 8 Hz), 13.22 (s, 1H, NH), 13.87 (s, 1H, NH). ^13^C–NMR (CDCl_3_, 100 MHz) ppm δ: 24.42, 31.43, 36.48, 39.49, 45.86, 108.34, 110.17, 116.15, 121.76, 123.91, 125.53, 126.93, 127.64, 131.06, 131.66, 133.19, 138.58, 150.68, 158.41, 165.91, 183.40. EI–MS *m*/*z* (C_24_H_24_BrClN_4_O_2_): calcd., 515; found: 515 [M]^+^, 517 [M+2H]^+^.

2-(2-(7-chloroquinolin-4-yl)hydrazineyl)-N’-(4-(2,3-dioxoindolin-1-yl)butyl)acetohydrazide (**11a**)

Brown solid, yield (0.24 g, 71%), m.p = 231–233 °C, FT–IR (KBr) cm^−1^ υ: 3405 (NH), 2935 (CH), 1728 (C=O), 1668 (C=O), 1605 (C=C_Ar_), 1577 (C=N), 1221(C-C), 813(C-Cl).^1^H–NMR (CDCl3,, 400 MHz) ppm δ: 1.78 (br.m, 4H, 2CH_2_), 3.46 (br.m, 4H, 2CH_2_), 3.79 (br.s, 2H, *N*-CH_2_), 7.44–8.27 (m, 8H, CH_Ar_), 8.79 (d, 1H, CH=N_Ar_, *J* = 8 Hz), 13.39 (s, 2H, 2NH), 13.65 (s, 2H, 2NH). ^13^C–NMR (CDCl_3_, 100 MHz) ppm δ: 22.78, 29.34, 29.80, 32.02, 59.91, 106.39, 123.67, 124.86, 127.80, 128.74, 129.14, 136.57, 152.24, 152.83, 171.88, 184.32. EI–MS *m*/*z* (C_23_H_23_ClN_6_O_3_): calcd., 466; found: 466 [M]^+^, 468 [M+2H]^+^.

2-((2-((7-chloroquinolin-4-yl)amino)ethyl)amino)-N’-(4-(2,3-dioxoindolin-1-yl)butyl)acetohydrazide (**11b**)

Reddish-orange solid, yield (0.27 g, 75%), m.p =224–226 °C, FT–IR (KBr) cm^−1^ υ: 3293 (NH), 2933 (CH), 1721(C=O), 1664 (C=O), 1605 (C=C_Ar_), 1577 (C=N), 1159 (C-C), 814 (C-Cl). ^1^H–NMR (CDCl_3_, 400 MHz) ppm δ: 1.84 (br.m, 4H, 2CH_2_), 3.38 (br.m, 4H, 2CH_2_), 3.42 (br.m, 4H, 2CH_2_), 3.79 (br.m, 2H, *N*-CH_2_), 7.48–8.12 (m, 8H, CH_Ar_), 8.77 (s, 1H, CH=N_Ar_), 13.70 (s, 2H, 2NH), 13.78 (s, 2H, 2NH). ^13^C–NMR (CDCl_3_, 100 MHz) ppm δ: 24.62, 24.99, 39.73, 53.50, 57.30, 102.79, 110.12, 120.49, 121.54, 123.94, 125.73, 127.14, 128.86, 129.15, 151.12, 162.62, 176.13, 184.31. EI–MS m/z (C_25_H_27_ClN_6_O_3_): calcd., 494; found: 494 [M]^+^, 496 [M+2H]^+^.

*2-((3-((7-chloroquinolin-4-yl)amino)propyl)amino)-N’-(4-(2,3-dioxoindolin-1-yl)butyl) acetohydrazide (* **11c** *)*

Brown solid, yield (0.25 g, 71%), m.p = 221–223 °C, FT–IR (KBr) cm^−1^ υ: 3343 (NH), 2933 (CH), 1705 (C=O), 1676 (C=O), 1609 (C=C_Ar_), 1566 (C=N), 1146(C-C), 745(C-Cl). ^1^H–NMR (CDCl_3_, 400 MHz) ppm δ: 1.83 (br.m, 6H, 3CH_2_), 2.11 (br.m, 2H, CH_2_), 3.30 (br.m, 2H, CH_2_), 3.63 (br.m, 2H, CH_2_), 3.74 (m, 2H, CH_2_), 3.86 (m, 2H, *N*-CH_2_), 6.89–8.07 (m, 8H, CH_Ar_), 8.78 (s, 1H, CH=N_Ar_), 13.67 (s, 2H, 2NH), 13.73 (s, 2H, 2NH). ^13^C–NMR (CDCl_3_, 100 MHz) ppm δ: 25.68, 31.65, 35.41, 36.95, 43.50, 53.95, 57.61, 103.50, 103.83, 110.81, 121.36, 122.55, 122.93, 124.53, 125.40, 128.05, 128.15, 128.59, 128.91, 130.99, 131.79, 137.16, 138.30, 149.91, 152.50, 152.94, 170.29, 182.65. EI–MS *m*/*z* (C_26_H_29_ClN_6_O_3_): calcd., 509; found: 509 [M]^+^, 511 [M+2H]^+^.

N’-(4-(5-bromo-2,3-dioxoindolin-1-yl)butyl)-2-(2-(7-chloroquinolin-4yl)hydrazineylacetohydrazide (**11d**)

Brown solid, yield (0.29 g, 75%), m.p = 232–234 °C, FT–IR (KBr) cm^−1^ υ: 3352 (NH), 2937 (CH), 1710 (C=O), 1657 (C=O), 1679 (C=CAr), 1564 (C=N), 1151(C-C), 807 (C-Cl), 645 (C-Br). ^1^H–NMR (CDCl_3_, 400 MHz) ppm δ: 1.85 (br.m, 4H, 2CH_2_), 3.77 (br.m,4H, 2CH_2_), 4.20 (br.s, 2H, *N*-CH_2_), 6.82–7.84 (m, 7H, CH_Ar_), 8.38 (s, 1H, CH=N_Ar_), 13.63 (s, 2H, 2NH), 13.71 (s, 2H, 2NH). ^13^C–NMR (CDCl_3_, 100 MHz) ppm δ: 24.27, 25.95, 29.69, 39.54, 62.88, 111.85, 116.86, 118.70, 119.36, 121.48, 128.34, 130.21, 131.45, 133.39, 140.77, 149.22, 157.75, 160.91, 178.31, 181.96. EI–MS *m*/*z* (C_23_H_22_BrClN_6_O_3_): calcd., 436; found: 545[M]^+^, 547[M+2H]^+^.

N’-(4-(5-bromo-2,3-dioxoindolin-1-yl)butyl)-2-((2-((7-chloroquinolin-4-yl)amino)ethyl)amino)acetohydrazide (**11e**)

Reddish-brown solid, yield (0.30 g, 73%), m.p = 227–230 °C, FT–IR (KBr) cm^−1^ υ: 3324 (NH), 2938 (CH), 1715 (C=O), 1654 (C=O), 1606 (C=C_Ar_), 1556 (C=N), 1174 (C-C), 815 (C-Cl), 679 (C-Br). ^1^H–NMR (CDCl_3_, 400 MHz) ppm δ: 1.85 (br.m, 4H, 2CH_2_), 3.17 (br.m, 2H, CH_2_), 3.34 (m, 4H, 2CH_2_), 3.49 (br.s, 2H, CH_2_), 3.79 (br.m, 2H, *N*-CH_2_), 7.46–8.18 (m, 7H, CH_Ar_), 8.77 (s, 1H, CH=N_Ar_), 13.52 (s, 2H, 2NH), 13.66 (s, 2H, 2NH). ^13^C–NMR (CDCl_3_, 100 MHz) ppm δ: 24.26, 25.78, 39.52, 51.09, 53.52, 111.79, 121.44, 125.07, 125.65, 128.50, 128.84, 136.80, 140.75, 143.11, 149.08, 150.71, 170.19, 184.52. EI–MS *m*/*z* (C_25_H_26_BrClN_6_O_3_): calcd., 573; found: 573 [M]^+^, 575[M+2H]^+^.

N’-(4-(5-bromo-2,3-dioxoindolin-1-yl)butyl)-2-((3-((7-chloroquinolin-4-yl)amino)propyl)amino) acetohydrazide (**11f**)

Violet solid, yield (0.26 g, 72%), m.p = 235–237 °C, FT–IR (KBr) cm^−1^ υ: 3365 (NH), 2937 (CH), 1731(C=O), 1651 (C=O), 1607 (C=C_Ar_), 1555 (C=N), 1291(C-C), 815 (C-Cl), 679(C-Br). ^1^H–NMR (CDCl_3_, 400 MHz) ppm δ: 1.92 (m, 6H, 3CH_2_), 3.16 (m, 2H, CH_2_), 3.48 (m, 4H, 2CH_2_), 3.78 (br.m, 2H, CH_2_), 4.31 (m, 2H, *N*-CH_2_), 6.83–8.18 (m, 7H, CH_Ar_), 8.77 (s, 1H, CH=N_Ar_), 13.41 (s, 2H, 2NH), 13.74 (s, 2H, 2NH). ^13^C–NMR (CDCl_3_, 100 MHz) ppm δ: 24.26, 25.86, 29.70, 39.52, 51.13, 53.58, 111.80, 121.45, 125.08, 125.66, 128.36, 128.92, 136.93, 140.75, 143.32, 148.87, 150.55, 157.75, 178.83, 183.90. EI–MS *m*/*z* (C_26_H_28_BrClN_6_O_3_): calcd., 587; found: 587 [M]^+^, 589 [M+2H]^+^.

### 3.3. Antibacterial Screening

In order to test the antibacterial activity of our conjugates, we performed preliminary screening for them by the agar-well diffusion method [44]. For all tested isolates, we began with 1.5 × 10^8^ CFU/mL (colony-forming units) (0.5 McFarland scale) inoculum. Under aseptic conditions, nutrient agar was poured into sterilized petri plates. Then, wells were made by sterile cork borer (6 mm in diameter) into agar plates after inoculation and 100 µL of each compound (40 mg/mL) was added to the wells. Finally, we incubated the plates at 37 °C for 20 h. We detected antibacterial activity by measuring the inhibition zone (including the well diameter) appearing after the incubation period. The DMSO was employed as a negative control. Positive control experiments were conducted to determine the sensitivity of the microorganisms: ampicillin (Sigma-Aldrich) and chloramphnicol (Sigma-Aldrich, St. Louis, MO, USA). The whole experiment was performed in triplicate.

#### 3.3.1. Minimum Inhibitory Concentration (MIC)

In this assay, the microtitre broth dilution method was adopted for all compounds. The MIC of the tested conjugates was determined by serially diluting them according to the Clinical and Laboratory Standards Institute (CLSI) guidelines [45]. To initiate, 1.5mL of compound stock solution in microcentrifuge tubes (Eppendorff) was prepared by dissolving powder to a final concentration of 20 mg/mL in dimethylsulphoxide (DMSO). Mueller-Hinton broth (Becton Dickinson, Sparks, MD, USA) was used to make serial dilutions from the stock solution ranging from 10 mg/mL to 0.004 mg/mL in 96-well microplates. A 24 h culture plate produced a bacterial suspension containing approximately 1.5108 CFU/mL (colony-forming units) (0.5 McFarland scale). One hundred liters of the prepared suspension was inoculated into each well. A sterility control well and a growth control well were investigated for each strain. The microtiter plates were incubated at 37 °C for 24 h. Then, after incubation at 37 °C, 40 μL of 0.4 mg/mL INT solution was added to each well as an indicator of microbial growth. After 30 min of incubating the plates at 37 °C, the MIC values were determined visually. The minimum inhibitory concentration was determined to be the lowest concentration of each extract that showed no visible growth. The MIC value was determined by taking the concentration that completely inhibited bacterial growth (the first clear well). To confirm activity, MIC values were determined in triplicate. Mueller-Hinton broth (Becton Dickinson, Sparks, MD, USA) was used in 96-well microplates to determine the final concentrations, ranging from 10 mg/mL (row A1) to 0.004 mg/mL (row A12). To ensure sterility, a negative control experiment was performed using only DMSO and MHB.

#### 3.3.2. Minimum Bactericidal Concentration (MBC)

The microtitre broth dilution method was also chosen for MBC determination. It was the lowest compound concentration, killing most (99.9%) of the bacterial inocula after 24 h incubation at 37 °C. The calculation of MBC was carried out using the Ozturk and Ercisli method [46]. It was performed on all conjugates tested. Under aseptic conditions, we took ten microliters from the MIC experiment well (MIC value) and two wells above the MIC value well and spread them on MHA plates. After 18–24 h of incubation at 37 °C, the colony count was determined. The MBC value was influenced by the concentration of sample that produced ten colonies. Each experiment was performed in triplicate.

#### 3.3.3. Time-Kill Assay

Only compounds with the lowest MBC/MIC ratio were considered [47]. It was carried out against *MRSA* clinical strain using the microplate method [48]. Samples and inoculums were prepared, 100 μL of sample in each well was filled with 100L of microbial broth at a concentration of 1.5108 CFU/mL (colony-forming units) (0.5 McFarland scale), grown in Mueller Hilton Broth (MHB). This resulted in a total volume of 200 μL of the culture in each well. The plates were then incubated at 37 °C for 18 h, with optical density monitored every 1 h at a wavelength of 600 nm. Turbidity was plotted against time on a graph. The obtained growth rate was investigated for bactericidal effects of the compounds.

### 3.4. Anti-Biofilm Assay

#### 3.4.1. Bacterial Culture Preparation

Three to five pure colonies of *MRSA* ATCC43300 were inoculated from the culture plate into 15 mL TSB, then the bacteria were revived in the shaker incubator at 200 rpm and at 37 °C for 18 to 24 h prior to the experiment so that they were preferably in their log phase of growth. Sterile TSB was used by autoclaving TSB at 121 °C, 1.5 atm for 20 min.

#### 3.4.2. Anti-Biofilm Assay

Bacterial suspension (1.5 × 10^8^ CFU/mL equivalent to UV absorbance reading of 0.1 with wavelength at 600 nm) was prepared in 15 mL from a 24 h bacterial culture. A 1:100 dilution was made in a separate centrifuge tube to obtain a 106 CFU/mL bacterial suspension. One hundred microliters of the diluted bacterial suspension was inoculated in TSB into the respective well of a new 96-well microplate then sterile distilled water was added to the 4 corners of the microplate to prevent evaporation of water from the test wells. Evaporation of water in test wells can interfere with the results. Alternatively, the microplate can be kept in a container with moist filter paper during the incubation. The plate was incubated at 37 °C in the incubator for 24 h. After incubation, the TSB broth was decanted completely from the microplate, the well was gently washed without disrupting the biomass formed, attaching on the bottom and wall of the wells with sterile phosphate buffer saline (PBS) 3 times. After that, 100 µL of freshly prepared sterile TSB broth (control well) was added in, and tested compounds suspended in TSB with test concentrations and TSB containing the vancomycin in test concentration. Vehicle control in DMSO is also needed to be aliquoted into appropriate wells as it is used as the diluent for the tested substances. The plate was covered with the lid and the plate was placed in incubator at 37 °C for 18 to 24 h. The 96-well plate was taken out of incubator and the TSB was slowly removed either by decanting or pipetting. The plate was rinsed with sterile double-distilled water and allowed to air-dry under the biosafety cabinet. The plates were turned upside down to speed up the process of drying. Dryness was ensured before moving on to the next step.

#### 3.4.3. Staining the Biofilm with Crystal Violet

One hundred microliters of aqueous crystal violet (1% *w*/*v*) was dispensed into the test wells to stain the bacterial cell walls for 10 to 15 min. The crystal violet was decanted onto clean disposable tissues. The test wells were rinsed three times with sterile double-distilled water and allowed to dry under the biosafety cabinet. Alternatively, the plate can be bathed subsequently with 3 dishes of water. Thirty percent (*v*/*v*) glacial acetic acid was dispensed in water to solubilize crystal violet and it was left standing for 15 min. It was ensured that there was clear violet solution with no visible residue in each of the test wells. Finally, the UV absorbance of all the wells at 570 nm was read. The anti-adherence activity of test substance and vancomycin were calculated using the following formula:

Anti-adherence activity% = 100 × ((Absorbance of control-Absorbance of test sample)/Absorbance of control)

### 3.5. Molecular Assay by Transmission Electron Microscopy (TEM)

Observation of TEM microscopy samples of *MRSA* was done by preparing slices of selected MRSA isolate. Bacterial samples were fixed for 1 h with pH 7.4 at 4 °C, immersed in 2.5% of glutaraldehyde (GA) and 0.1 M of sodium cacodylate buffer, then washed in cacodylate buffer. All bacterial samples were double-fixed in cacodylate buffer containing 1% of osmium tetroxide (OsO_4_) for 90 min at room temperature. Furthermore, we dehydrated the samples in acetone and the dehydrated cells were embedded in Epon-Araldite (502 kit, Pelco, CA, USA). After that, 500–1000 nm sections of bacterial samples were obtained using a Leica EM UC6 ultra-microtome (Wetzlar, Germany) mounted on glass slides and stained with 1% toluidine blue stain. All sample sections were scanned and investigated using a JEM 1011 (JEOL) electron microscope set to 80 kv [49].

### 3.6. Molecular Modeling

The targeted enzyme was downloaded from Protein Data Bank along with its bounded co-crystallized ligand (PDB:4DKI). Preparation of the downloaded protein was performed following the literature [50]

For optimization of the synthesized compounds, all structures were copied as smiles from chem-draw, bonds and restraints were enabled, calculating of partial charges was performed and compounds were saved upon energy minimization in mol2 format.

The protocol for docking was performed using MOE software [51] following the reported procedures [41] 

## 4. Conclusions

New conjugates **10a**–**f** and **11a**–**f** bearing quinoline and isatin scaffolds were prepared in good yields. The structures of the conjugates were established based on spectroscopic data. The antibacterial activity for all synthesized conjugates was evaluated and displayed activity higher than the first line antibiotics ampicilin and chloramphnicol used in this study. In addition, the synthesized conjugates exhibited good to excellent bactericidal and anti-biofilm activity. Moreover, the TEM study proved their mechanism of action where a damage in both cell wall and membrane was observed with the appearance of damaged vacuoles in cell cytoplasm. Molecular docking studies were performed on the prepared compounds **10a**–**f** and **11a**–**f,** which showed promising binding affinity for the transpeptidase receptor active site in *MRSA* and the main interactions between the synthesized ligands and active site involved THR 600, HIS583, GLN521, SER462 and TYR446. The range of binding affinity was −8.098 to −6.501 and the RMSD values ranged from 1.184 to 2.134 A. Compounds **10a**–**f** and **11a**–**f** were subjected to in silico assessment for their pharmacokinetic properties; all the assessed compounds were considered as “drug-like” molecules with promising bioavailability.

## Figures and Tables

**Table 1:** Spectral data for conjugates **10a**–**f** and **11a**–**f**.

	FT-IR υ (cm^−1^)					^1^H-NMR δ (ppm)			^13^C-NMR δ (ppm)			
Com.	NH	υ _(CH)_ Aliphatic	υ _(C=O)_ Isatin	υ _(N-C=O)_ Isatin	υ _(C=N)_ Quinoline	NH-CH_2_ Quinoline	N-CH_2_ Isatin	CH=N_Ar_ Quinoline	CH=N_Ar_ Quinoline	N-C=O Isatin	C=O (C-3) Isatin	C=O (C-2) hydrazide
**10a**	3374	2930	1710	1675	1562	3.79		8.54	153.10	167.35	183.20	
**10b**	3319	2936	1703	1665	1577	3.78	3.88	8.77	150.61	158.17	183.40	--------
**10c**	3397	2931	1713	1654	1559	3.77	4.28	8.55	155.49	162.34	184.53	--------
**10d**	3360	2933	1732	1655	1587	3.26	3.91	8.82	158.24	162.55	183.29	--------
**10e**	3312	2949	1731	1657	1555	3.45	3.81	8.81	151.02	167.17	181.95	--------
**10f**	3343	2934	1705	1653	1587	3.75	3.84	8.65	158.41	165.41	183.40	--------
**11a**	3405	2935	1718	1668	1577	3.46	3.79	8.79	152.24	152.82	184.52	171.88
**11b**	3297	2933	1721	1664	1577	3.42	3.79	8.77	151.12	162.62	184.31	176.13
**11c**	3343	2933	1705	1676	1566	3.47	3.86	8.78	152.50	152.94	182.65	170.29
**11d**	3352	2937	1710	1657	1564	3.77	4.20	8.38	157.75	160.91	181.96	178.31
**11e**	3324	2938	1715	1654	1556	3.49	3.79	8.77	149.08	150.71	184.52	170.14
**11f**	3365	2937	1731	1651	1555	3.78	4.31	8.77	150.55	157.75	183.90	178.83

**Table 2:** Antibacterial activity of conjugates **10a**–**f** and **11a**–**f** against *MRSA* clinical isolate.

Conjugates	Zone of Inhibition ZOI (mm) ^1^	*MRSA*			MRSA ATCC 43300		
		Minimum Bactericidal Concentration MBC ^2^	Minimum Inhibitory Concentration MIC ^3^	MBC/MIC	MBC	MIC	MBC/MIC
**1a**	13 ± 1	10	2.5	4(+)	10	5	2(+)
**1b**	13.3 ± 0.6	10	2.5	4(+)	10	5	2(+)
**4**	12.3 ± 0.6	20	2.5	8(+)	20	5	4(+)
**10a**	24.31 ± 0.60	0.05	0.006	8 (+)	0.05	0.025	2(+)
**10b**	25.00 ± 1.00	0.05	0.0125	4 (+)	0.05	0.025	2(+)
**10c**	23.00 ± 1.00	0.05	0.025	2(+)	0.05	0.025	2(+)
**10d**	23.75 ± 0.60	0.05	0.025	2(+)	0.05	0.0125	4 (+)
**10e**	24.73 ± 0.60	0.05	0.025	2(+)	0.05	0.025	2(+)
**10f**	24.78 ± 0.60	0.05	0.025	2(+)	0.05	0.0125	4 (+)
**11a**	47.33 ± 0.60	0.625	0.156	4 (+)	0.156	0.08	2 (+)
**11b**	24.00 ± 1.00	2.5	1.25	2(+)	5	2.5	2 (+)
**11c**	30.72 ± 1.00	0.312	0.156	2(+)	0.625	0.312	2 (+)
**11d**	20.00 ± 1.00	2.5	1.25	2(+)	5	2.5	2 (+)
**11e**	18.31 ± 0.60	2.5	1.25	2(+)	5	2.5	2 (+)
**11f**	16.34 ± 0.60	5	1.25	4 (+)	5	2.5	2 (+)
Ampicilin	14.77 ± 0.60	6.25	3.13	2(+)	6.25	1.56	4 (+)
Chloramphnicol	14.32 ± 0.60	3.13	0.78	4(+)	12.5	8	1.6(+)

^1^: Zone of inhibition, ^2^: Minimum bactericidal concentration, ^3^: minimum inhibitory concentration.

**Table 3:** Antibacterial activity of conjugates **10a**–**f** and **11a**–**f** against *Streptococcus mutans* clinical isolate.

Conjugates	Zone of Inhibition ZOI (mm) ^1^	*Streptococcus mutans*			*Streptococcus mutans* ATCC 35668		
		Minimum Bactericidal Concentration MBC ^2^	Minimum Inhibitory Concentration MIC ^3^	MBC/MIC	MBC	MIC	MBC/MIC
**1a**	14 ± 1	10	1.25	8(+)	10	2.5	4(+)
**1b**	13 ± 1	5	1.25	4(+)	10	2.5	4(+)
**4**	14.3 ± 0.6	20	2.5	8(+)	20	5	4(+)
**10a**	23 ± 1	0.0008	0.0004	1.9 (+)	0.003	0.0015	2 (+)
**10b**	20.3 ± 0.6	0.003	0.0008	4 (+)	0.003	0.0008	4 (+)
**10c**	18 ± 1	0.0016	0.0008	2 (+)	0.003	0.0015	2 (+)
**10d**	17.3 ± 0.6	0.0004	0.0002	2 (+)	0.0008	0.0004	2 (+)
**10e**	20.7 ± 0.6	0.0004	0.0002	2 (+)	0.0004	0.0002	2 (+)
**10f**	20 ± 1	0.0004	0.0002	2 (+)	0.0004	0.0002	2 (+)
**11a**	46.3 ± 1	0.156	0.08	1.9 (+)	0.625	0.312	2 (+)
**11b**	22 ± 1	1.25	0.312	4 (+)	2.5	1.25	2(+)
**11c**	24 ± 1	0.625	0.156	4 (+)	1.25	0.625	2 (+)
**11d**	21.7 ± 0.6	0.625	0.156	4 (+)	1.25	0.625	2 (+)
**11e**	20.3 ± 0.6	0.312	0.156	2 (+)	0.156	0.08	2 (+)
**11f**	14.3 ± 0.6	5	2.5	2 (+)	10	5	2(+)
Ampicilin	12 ± 1	3.13	1.565	2 (+)	6.25	3.13	2(+)
Chloramphnicol	12.7 ± 0.6	6.25	1.56	4 (+)	12.5	6.25	2(+)

^1^: Zone of inhibition, ^2^: Minimum bactericidal concentration, ^3^: minimum inhibitory concentration.

**Table 4:** Antibacterial activity of conjugates **10a**–**f** and **11a**–**f** against *Klebsiella pneumoniae* clinical isolate.

Conjugates	Zone of Inhibition ZOI (mm) ^1^	*Klebsiella pneumoniae*			*Klebsiella pneumoniae* ATCC 700603		
		Minimum Bactericidal Concentration MBC ^2^	Minimum Inhibitory Concentration MIC ^3^	MBC/MIC	MBC	MIC	MBC/MIC
**1a**	13.7 ± 0.6	5	1.25	4(+)	10	5	2
**1b**	12 ± 1	10	2.5	4(+)	10	5	2
**4**	11 ± 1	40	5	8(+)	20	5	4
**10a**	23 ± 1	0.003	0.0008	4(+)	0.006	0.003	2(+)
**10b**	17 ± 1	0.0125	0.006	2(+)	0.025	0.0125	2(+)
**10c**	22 ± 1	0.625	0.156	4(+)	0.625	0.312	2(+)
**10d**	19 ± 1	0.0125	0.006	2(+)	0.0125	0.006	2(+)
**10e**	21 ± 1	0.003	0.0008	4(+)	0.006	0.003	2(+)
**10f**	21.3 ± 0.6	0.006	0.003	2(+)	0.0125	0.006	2(+)
**11a**	41.6 ± 0.6	0.156	0.04	4 (+)	0.312	0.156	2(+)
**11b**	23 ± 1	1.25	0.625	2(+)	2.5	1.25	2(+)
**11c**	25.3 ± 0.6	0.312	0.08	4(+)	0.625	0.312	2(+)
**11d**	23.7 ± 0.6	0.625	0.156	4(+)	1.25	0.625	2(+)
**11e**	25 ± 1	0.312	0.08	4(+)	0.312	0.156	2(+)
**11f**	16 ± 1	5	1.25	4(+)	5	2.5	2(+)
Ampicilin	14.3 ± 0.6	8	4	2(+)	6.25	3.125	2(+)
Chloramphnicol	8.7 ± 0.6	3.13	1.565	2 (+)	6.25	1.56	4 (+)

^1^: Zone of inhibition, ^2^: Minimum bactericidal concentration, ^3^: minimum inhibitory concentration.

**Table 5:** Antibacterial activity of conjugates **10a**–**f** and **11a**–**f** against the *Serratia marcescens* clinical isolate.

Conjugates	Zone of Inhibition ZOI (mm) ^1^	*Serratia marcescens*			*Serratia marcescens* ATCC13880		
		Minimum Bactericidal Concentration MBC ^2^	Minimum Inhibitory Concentration MIC ^3^	MBC/MIC	MBC	MIC	MBC/MIC
**1a**	14.7 ± 0.6	5	2.5	2(+)	10	5	2(+)
**1b**	14.5 ± 0.6	5	1.25	4(+)	5	2.5	2(+)
**4**	13.3 ± 0.6	20	5	4(+)	20	10	2(+)
**10a**	21.7 ± 0.6	0.0008	0.0004	2(+)	0.0018	0.0008	2(+)
**10b**	26.3 ± 0.6	0.003	0.0008	4(+)	0.006	0.003	2(+)
**10c**	28 ± 1	0.0004	0.0002	2(+)	0.0008	0.0004	2(+)
**10d**	23.7 ± 0.6	1.25	0.62	2(+)	0.006	0.003	2(+)
**10e**	22.7 ± 0.6	0.05	0.0125	4(+)	0.05	0.025	2(+)
**10f**	28 ± 1	0.003	0.0008	4(+)	0.003	0.0008	4(+)
**11a**	43 ± 1	0.08	0.04	2(+)	0.156	0.08	2(+)
**11b**	15.3 ± 0.6	1.25	0.312	4(+)	2.5	1.25	2(+)
**11c**	32.3 ± 0.6	1.25	0.312	4(+)	2.5	1.25	2(+)
**11d**	23.3 ± 0.6	5	1.25	4(+)	5	2.5	2(+)
**11e**	17.3 ± 0.6	5	1.25	4(+)	5	2.5	2(+)
**11f**	10.7 ± 0.6	160	40	4(+)	160	80	2(+)
*Ampicilin*	8.7 ± 0.6	12.5	6.25	2(+)	6.25	1.56	4 (+)
*Chloramphnicol*	13.3 ± 0.6	3.13	1.565	2 (+)	3.13	1.565	2 (+)

^1^: Zone of inhibition, ^2^: Minimum bactericidal concentration, ^3^: minimum inhibitory concentration.

**Table 6:** MIC, MBC and MBC/MIC ratio for **10a**–**f** and **11a**–**f** against MRSA.

Conjugates	MBC	MIC	MBC/MIC Ratio
**10a**	0.025	0.006	4 (+)
**10b**	0.05	0.0125	4(+)
**10c**	0.05	0.025	2(+)
**10d**	0.05	0.025	2(+)
**10e**	0.05	0.025	2(+)
**10f**	0.05	0.025	2(+)
**11a**	0.625	0.156	4 (+)
**11b**	2.5	1.25	2(+)
**11c**	0.312	0.156	2(+)
**11d**	2.5	1.25	2(+)
**11e**	2.5	1.25	2(+)
**11f**	5	1.25	4(+)

**Table 7:** Anti-biofilm assay of conjugate **10** towards *MRSA* ATCC 43300. Experiment evaluated based on quadruplicate results with standard deviation. (n = 4).

Concentration (mg/mL)	10a	10b	10c	10d	10e	10f
10	79.4 ± 0.22	65.4 ± 0.05	68.3 ± 0.07	71.8 ± 0.06	77.4 ± 0.07	62.9 ± 0.07
5	75.5 ± 0.11	59.8 ± 0.08	61.3 ± 0.08	65.3 ± 0.05	70.9 ± 0.06	58.8 ± 0.07
2.5	57.2 ± 0.07	48.8 ± 0.07	46.8 ± 0.05	43.8 ± 0.07	61.4 ± 0.07	43.9 ± 0.07
1.25	38.5 ± 0.07	33.4 ± 0.05	32.8 ± 0.06	31.7 ± 0.06	46.4 ± 0.07	34.6 ± 0.08

**Table 8:** Anti-biofilm assay of conjugates of **11** towards *MRSA* ATCC 43300. Experiment evaluated based on quadruplicate results with standard deviation. (n = 4).

Concentration (mg/mL)	11a	11b	11c	11d	11e	11f
10	83.6 ± 0.11	72.7 ± 0.06	77.7 ± 0.08	67.4 ± 0.11	58.8 ± 0.08	50.2 ± 0.11
5	78.4 ± 0.08	58.6 ± 0.08	62.5 ± 0.08	54.2 ± 0.11	47.5 ± 0.13	41.9 ± 0.06
2.5	62.6 ± 0.09	43.3 ± 0.08	53.8 ± 0.08	39.3 ± 0.11	32.3 ± 0.11	24.7 ± 0.14
1.25	48.8 ± 0.06	34.7 ± 0.07	44.4 ± 0.09	28.5 ± 0.09	24.9 ± 0.09	16.4 ± 0.11

**Table 9:** Molecular modeling data regarding binding affinity, RMSD values and receptor residues involved in interactions.

Compound No.	Binding Affinity	Root Mean Square Deviation (RMSD)	Amino Acids Involved in Interactions at the Active Site
**10a**	−7.181	1.674	THR600(H-donor), HIS583(H-acceptor), THR600(*pi*-H)
**10b**	−6.501	1.616	THR 600(H-donor), HIS583(H-acceptor), THR600(*pi*-H)
**10c**	−6.805	1.873	GLN521(H-donor), GLU447(*pi*-H)
**10d**	−6.992	1.498	MET641(H-acceptor)
**10e**	−7.191	1.184	THR 600(H-donor), THR 600(H-acceptor), THR 600(H-acceptor)
**10f**	−7.171	2.030	SER462(H-acceptor), SER598(H-acceptor), LYS639(*pi*-H)
**11a**	−7.254	1.464	THR 600(H-donor), THR 600(H-acceptor), ASN464(H-donor), ASN464(H-acceptor)
**11b**	−7.323	1.300	THR 600(H-donor), GLN521(*pi*-H)
**11c**	−8.098	1.784	GLU523(H-donor), TYR519(H-donor), GLN521 (H-donor), GLU447(H-donor), SER403(H-acceptor), SER462(*pi*-H)
**11d**	−7.115	2.134	TYR446 (*pi*-H)
**11e**	−7.444	1.987	THR 600(H-donor), TYR446(*pi*-H), TYR446(*pi*-H)
**11f**	−7.741	1.825	THR 600(H-acceptor), THR 600(H-acceptor), GLY640(H-donor)
**RB6**	−6.9862	1.0736	THR 600(H-acceptor), ASN464(H-acceptor), GLU447(H-donor), TYR519(H-donor)

**Table 10:** Results recorded by compounds **10a**–**f** and **11a**–**f** upon in silico pharmacokinetic screening.

**Molecule**	**MWt**	**HBA**	**HBD**	**TPSA**	**iLog*P***	**GI Absorption**	**BBB Score**	**Drug Likeness Score**	**Bioavailability Score**
**10a**	394.85	4	2	74.33	2.98	High	3.84	0.53	0.55
**10b**	422.91	4	2	74.33	3.36	High	3.39	0.47	0.55
**10c**	436.93	4	2	74.33	3.55	High	3.76	0.47	0.55
**10d**	473.75	4	2	74.33	3.27	High	4.01	0.31	0.55
**10e**	501.8	4	2	74.33	3.61	High	3.54	0.26	0.55
**10f**	515.83	4	2	74.33	3.85	High	3.89	0.25	0.55
**11a**	466.92	6	4	115.46	2.41	High	2.36	0.66	0.55
**11b**	494.97	6	4	115.46	3.1	High	2.34	0.59	0.55
**11c**	509	6	4	115.46	3.15	High	2.30	0.57	0.55
**11d**	545.82	6	4	115.46	2.53	High	2.31	0.44	0.55
**11e**	573.87	6	4	115.46	2.69	High	2.30	0.39	0.55
**11f**	587.9	6	4	115.46	3.45	High	2.25	0.37	0.55

## Supplementary Materials

The following supporting information can be downloaded at: https://www.mdpi.com/article/10.3390/antibiotics11111507/s1, Figure S1–S4 displayed the zone of inhibition for conjugates **10** and **11** against Gram-positive and Gram-negative bacteria clinical isolates; Figures S5–S7 presented the antibiofilm assay for conjugates **10** and **11**.
