# Supplementary material for: Synthesis, Biocidal and Antibiofilm Activities of New Isatin–Quinoline Conjugates against Multidrug-Resistant Bacterial Pathogens along with Their In Silico Screening
Source: Antibiotics (Basel). 2022 Oct 28;11(11):1507. doi: 10.3390/antibiotics11111507 (PMC9686684; doi:10.3390/antibiotics11111507)
Supplement: Supplementary file 1 [file antibiotics-11-01507-s001.zip › antibiotics-1981113-supplementary.pdf]

Supporting information

Synthesis, biocidal and antibiofilm activities of new isatin–quinoline conjugates against multidrug-resistant bacterial pathogens along with their *in silico* screening

Elshaymaa I. Elmongy [1], Abdullah A. S. Ahmed [2], Ibrahim El Tantawy El Sayed[*2], Ghady Fathy [2], Hanem M. Awad [3], Ayah Usama Salman[4], Mohamed A. Hamed [5]

[1] Department of Pharmaceutical Sciences, College of Pharmacy, Princess Nourah bint Abdulrahman University, Riyadh 84428, Saudi Arabia
[2] Chemistry Department, Faculty of Science, Menoufia University, Shebin El-Kom 32511, Egypt
[3] Department of Tanning Materials and Leather Technology, National Research Centre, Dokki, Giza, Egypt
[4]Department of Botany and Microbiology, Faculty of Science, Menoufia University, Shebin El-Kom 32511, Egypt
[5] Chemistry Department, Faculty of Science, Tanta University, Tanta, 31511 Egypt.

* Correspondence: Ibrahim El Tantawy El Sayed
Chemistry Department, Faculty of Science, Menoufia University, Shebin El-Kom 32511, Egypt,
E-mail: ibrahimtantawy@yahoo.co.uk

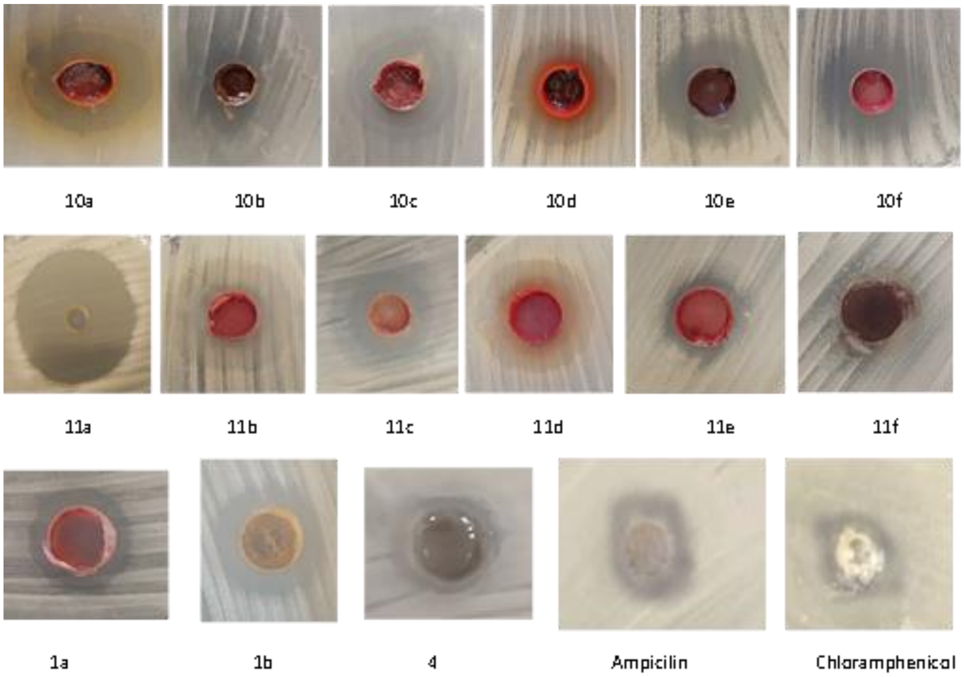

Figure S1: Inhibition zone of **10a-f** and **11a-f** against *MRSA* clinical isolate

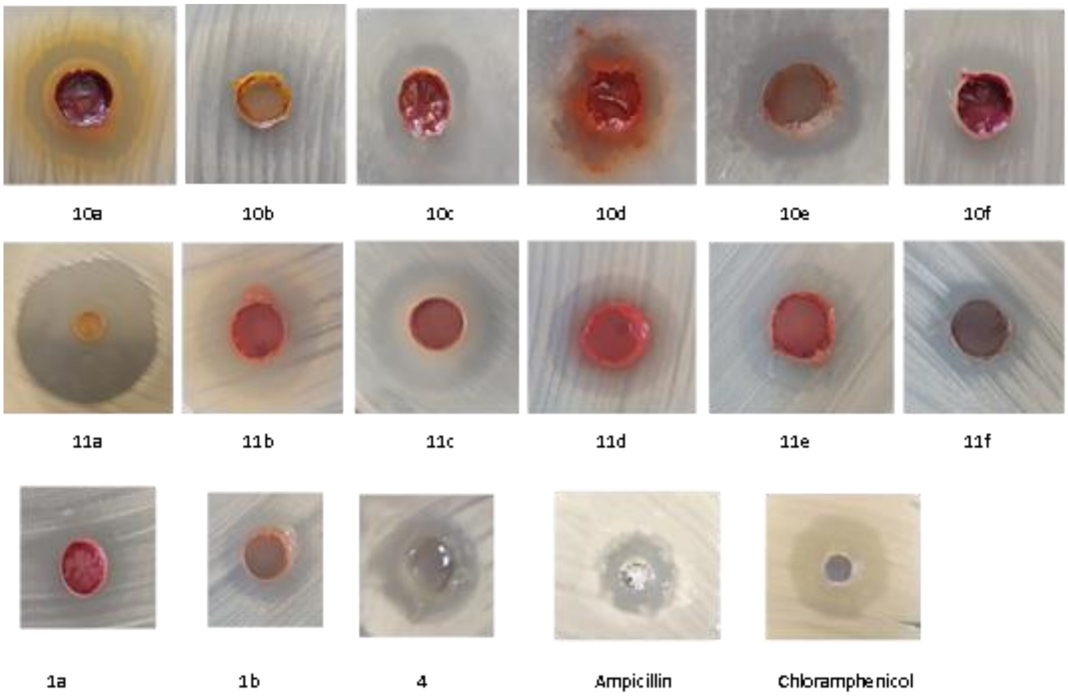

Figure S2: Inhibition zone of **10a-f** and **11a-f** against *Streptococcus mutans* clinical isolate

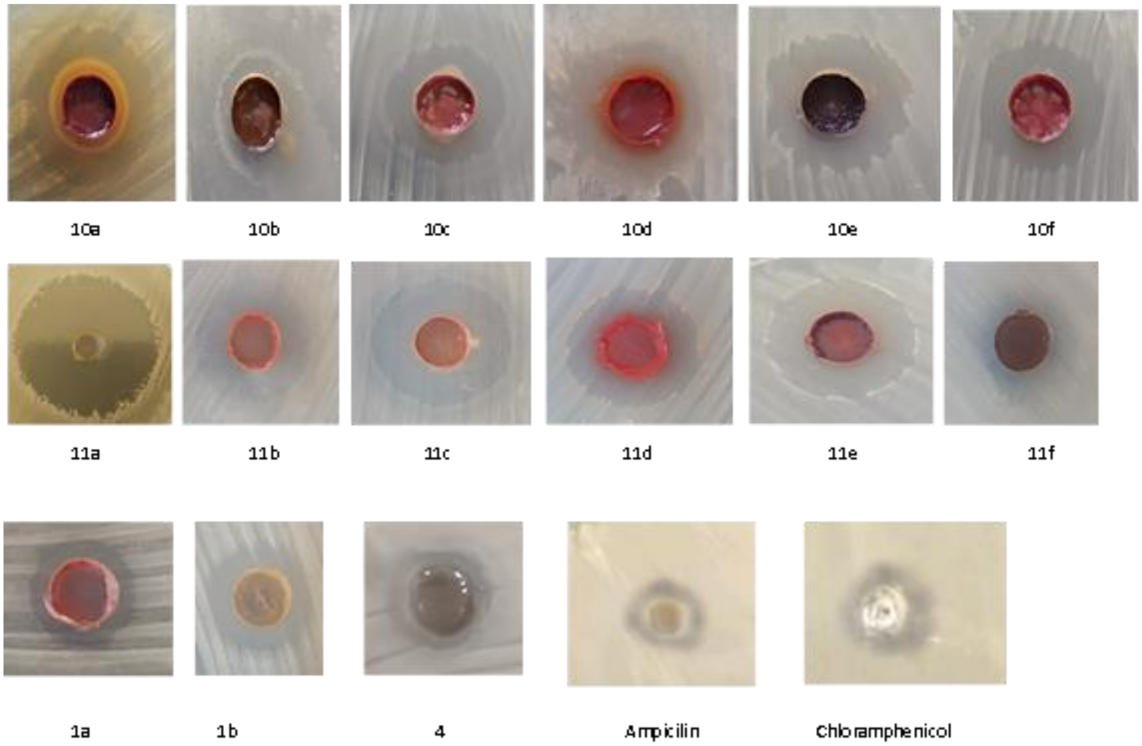

Figure S3: Inhibition zone of **10a-f** and **11a-f** against *Klebsiella pneumonia* clinical isolate

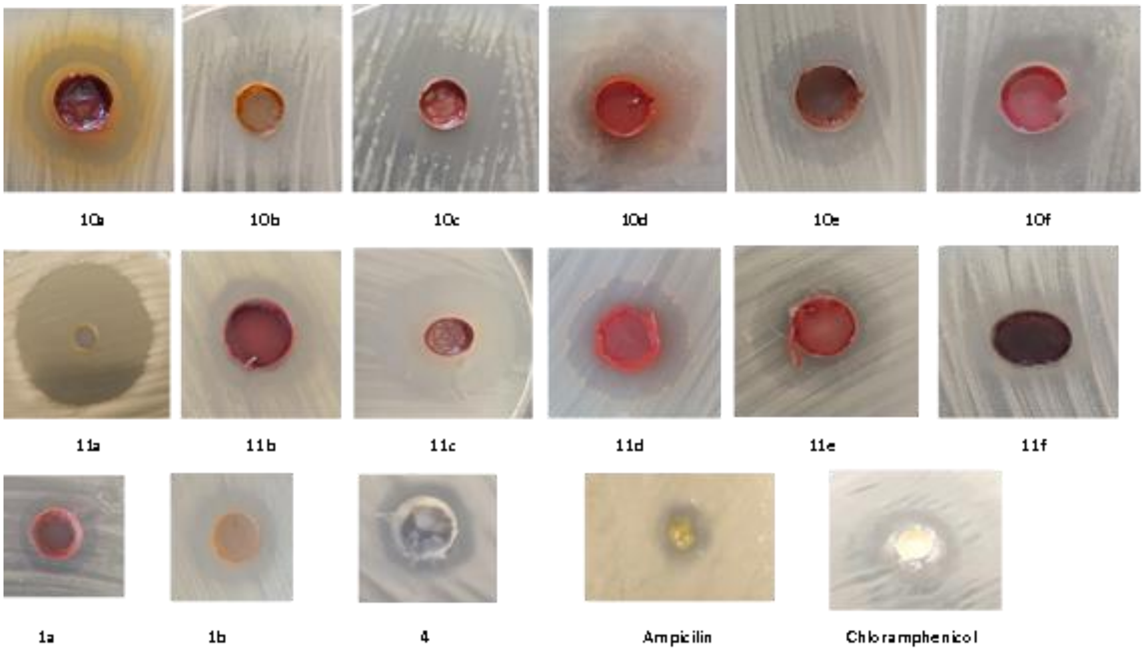

Figure S4: Inhibition zone of **10a-f** and **11a-f** against *Serratia marcescens* clinical isolate

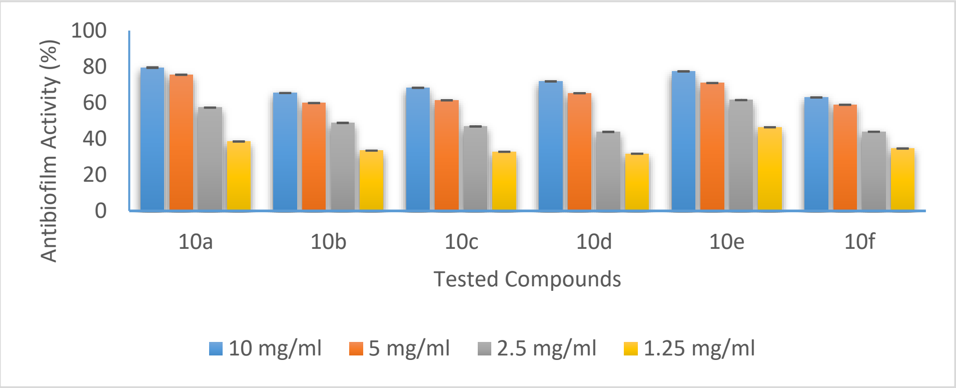

Figure S5: Anti-biofilm assay of conjugates **10a-f** against *MRSA* ATCC 43300. Experiment evaluated based on quadruplicate results with standard deviation. (n = 4).

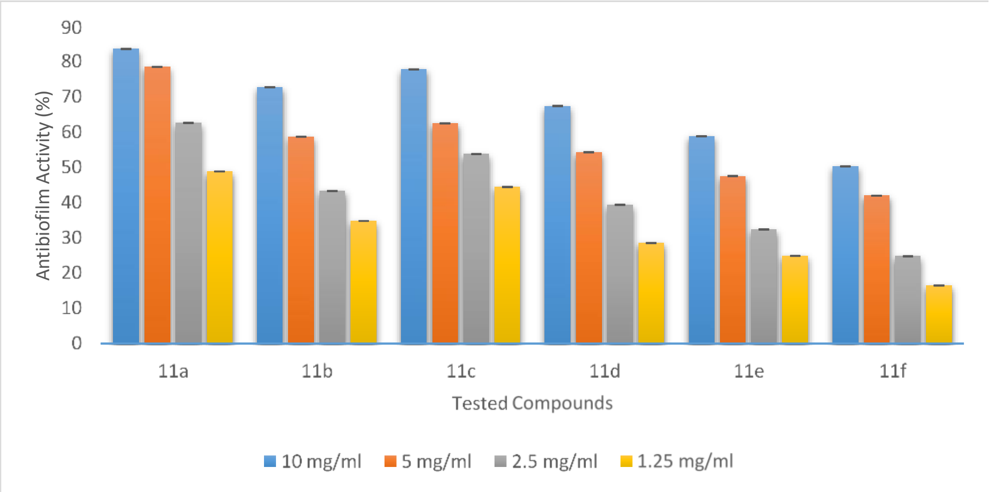

Figure S6. Anti-biofilm assay of series **11a-f** towards *MRSA* ATCC 43300. Experiment evaluated based on quadruplicate results with standard deviation. (n = 4).

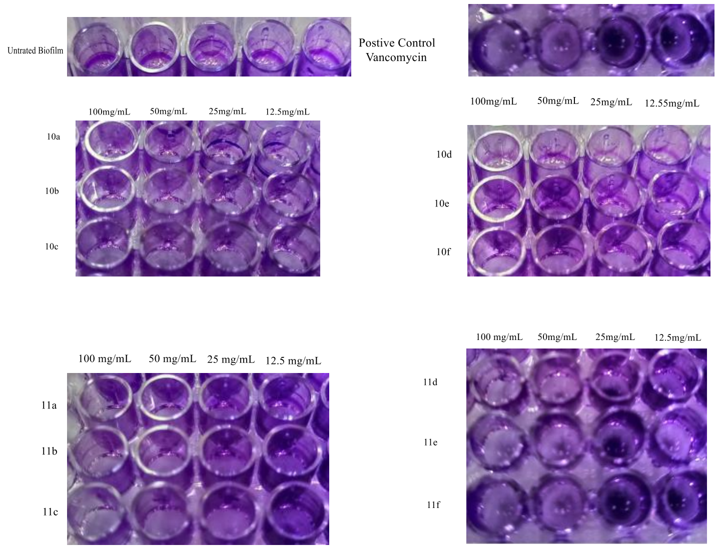

Figure S7: anti-biofilm screening for conjugates **10** and **11** with different concentration against *MRSA* ATCC 43300.
